# Identification of pathogen genomic variants through an integrated pipeline

**DOI:** 10.1186/1471-2105-15-63

**Published:** 2014-03-03

**Authors:** Micah J Manary, Suriya S Singhakul, Erika L Flannery, Selina ER Bopp, Victoria C Corey, Andrew Taylor Bright, Case W McNamara, John R Walker, Elizabeth A Winzeler

**Affiliations:** 1 Department of Pediatrics, University of California, San Diego, School of Medicine, 9500 Gilman Drive 0741, La Jolla, California 92093, USA; 2 Biomedical Sciences Program, University of California, San Diego, 9500 Gilman Drive, La Jolla, California 92093, USA; 3 Immunology and Infectious Diseases, Harvard School of Public Health, 665 Huntington Avenue, Boston, Massachusetts 02115, USA; 4 Genomics Institute of the Novartis Research Foundation, 10675 John Jay Hopkins Drive, San Diego, California 92121, USA

**Keywords:** Malaria, Sequencing, Genome, Polymorphism, Variant

## Abstract

**Background:**

Whole-genome sequencing represents a powerful experimental tool for pathogen research. We present methods for the analysis of small eukaryotic genomes, including a streamlined system (called Platypus) for finding single nucleotide and copy number variants as well as recombination events.

**Results:**

We have validated our pipeline using four sets of *Plasmodium falciparum* drug resistant data containing 26 clones from 3D7 and Dd2 background strains, identifying an average of 11 single nucleotide variants per clone. We also identify 8 copy number variants with contributions to resistance, and report for the first time that all analyzed amplification events are in tandem.

**Conclusions:**

The Platypus pipeline provides malaria researchers with a powerful tool to analyze short read sequencing data. It provides an accurate way to detect SNVs using known software packages, and a novel methodology for detection of CNVs, though it does not currently support detection of small indels. We have validated that the pipeline detects known SNVs in a variety of samples while filtering out spurious data. We bundle the methods into a freely available package.

## Background

The detection of single nucleotide and copy number variants (SNVs and CNVs) conferring resistance to drug and vaccine candidates provides researchers with a powerful tool to choose the best combination of agents to treat infectious diseases such as malaria in specific regions, to study pathogen population dynamics and transmission, as well as to engineer new treatments that cannot be easily evaded. In addition, in organisms in which genetic complementation or backcrosses may be difficult or time consuming, whole genome sequencing (WGS) offers an opportunity to determine if second-site mutations may have been inadvertently introduced after transfection or transformation, and contribute to an observed phenotype.

With the reduction in price and increased power of current short-read high-throughput WGS methods and the wide dispersal of a variety of sequencing platforms and accompanying support, full genome sequence data is now relatively easy to generate. Recent advances in the algorithmic and programmatic analysis of WGS data have led to a number of standards, especially the use of the Genome Analysis Toolkit (GATK) [1], being used in the analyses of human genomic data to detect SNVs and CNVs. However, there are opportunities for more comprehensive analyses of the genomes of simpler eukaryotes such as the ~23.5 Mb genome of *Plasmodium falciparum*, the apicomplexan parasite and etiological agent of human malaria, which has also served as a model for eukaryotic pathogen genomics since the completion and full assembly of its genome sequence in 2002 [2]. Full genome sequencing at 30-40X coverage is now readily achieved [3-6]. Such coverage allows for the identification of recombination events, the description of SNVs in sequences other than in the exomes, and the detection of small structural variants, including short-length insertion or deletion events. *P. falciparum* is responsible for up to a million deaths annually [7], and although its haploid genome is worthy of investigation for this reason alone, it also serves as an ideal test system because heterozygous calls generally do not need to be considered in sequence analysis validation (although mixed infections are a real concern) and a fully assembled reference genome is available [2]. Furthermore, the parasite can be sub-cloned and readily cultured *in vitro* within white-cell depleted, anucleated human erythrocytes [8], mitigating host DNA contamination.

In this manuscript, we introduce a validated pipeline for the comprehensive analysis of short-read WGS data in *Plasmodium* spp.. The pipeline, which can be readily adapted to other small eukaryotes, integrates well-known alignment tools and custom filtration options so that SNV or structural variant data can be easily generated and understood. We believe that the pipeline will work well, once adapted, with species of any ploidy (indeed, it has been used already in *Arabidopsis* analysis) and genomes of size up to 75 Mbp have been tested. As well, we introduce improved algorithms for utilizing depth of coverage to call CNVs, improving on current GC bias normalization methods [9]. This pipeline is implemented in a stand-alone program called “Platypus”, for open distribution and collaboration among research groups. We validate the pipeline using data from 26 *P. falciparum* samples with known SNVs and CNVs (Table 1), demonstrating both its accuracy and precision. This pipeline should allow those generating WGS data to not only find all SNVs and structural variants detected by other methods (as well as novel ones) but to eliminate all or almost all false positives, reducing ambiguity and potentially allowing WGS to substitute for complementation, Southern blotting, or other genetic methods designed to link phenotype to genotype.

**Table 1:** Whole-genome sequencing statistics

**Experiment ID**	**Data source**	**Background strain**	**# of genomes**	**Resistance**	**Gene conferring resistance**
Dd2 (parent)	[6,10-12]	n/a	1	Chloroquine, Mefloquine, Pyrimethamine	n/a
3D7 (parent)	[12]	n/a	1	Sulfadoxine	n/a
KAD707 and 458	[12]	Dd2	4	Imidazolopiperazine	*pfcarl*
CladoR	[10], this study	Dd2	3	Cladosporin	*pfkrs1*
NITD609 (KAE609)	[6,11]	Dd2	3	Spiroindolone	*pfatp4*
NITD678	[6,11]	Dd2	3	Spiroindolone	*pfatp4*
Evo	[6]	3D7	14	Atovaquone	*mal_mito_3*

Whole genome sequencing data acquisition statistics from each of the experiments used to validate the pipeline. Note that all of the lines with variants to be called were sub-cloned before sequencing and were sequenced as 70 bp paired end reads, except the KAD (imidazolopiperazines resistant) lines, which were sequenced as 60 bp single end reads. Library preparation details for sequences obtained from previous studies are available in their respective manuscripts. Library preparations for sequences obtained for this study (those resistant to cladosporin), are as follows: 200 bp fragments with 70 bp reads were prepared according to the manufacturers instructions using the Illumina NexTera XT sample preparation kit with accompanying primers and sheared using a Covaris E220x machine.

n/a – not applicable.

## Implementation

Current genotyping programs are generally designed to be conservative and as a consequence, return a large number of false positive variant calls. These programs, including GATK [1] and the sequence/alignment map toolbox (SAMTools) [13], typically allow the user to set a number of stringency filters such as the quality of the read alignment or bias towards a specific strand, that can theoretically be used to separate false from true positives. However, the actual threshold values for each filter are not pre-determined, and as such, it is left to the researcher to decide how to best utilize each metric, creating barriers for the novice user. Thus, we set out to create a set of empirically-derived filters for *Plasmodium* WGS data that could be used as a reference point for future SNV analyses.

To identify a robust set of filtering parameters we began with a list of 15,145 known SNVs identified using traditional Sanger resequencing of Dd2 to 7X coverage [14] and deposited in PlasmoDB (http://plasmodb.org)[15]. These distinguish the multidrug-resistant *P. falciparum* laboratory Indochina strain, Dd2, from the African drug-sensitive reference strain, 3D7. We then compared a *P. falciparum* Dd2 strain WGS short-read sequence obtained in our lab to the *P. falciparum* reference (3D7 strain) sequence. Our Dd2 sequence was generated with 70 bp paired-end reads on an Illumina Genome Analyzer II to a mean of 31X coverage with 96.4% of bases being covered by 5 reads or more. We considered the 15,145 curated SNVs to be true positives. All other SNVs detected were considered false positives, although it is likely that some of the novel SNVs are indeed true genetic differences (genetic diversity, especially in the subtelomeric regions, is extremely high approaching 90% diversity in at least one base position between field samples) [16]. We then worked to identify a set of filtering parameters, which would have the sensitivity to detect at least 90% of the known SNVs, while eliminating as many ‘novel’ SNVs as possible.

Because the entire mathematical domain of all commonly used filtering parameters (17 characteristics of SNVs and their combinations, see Table 2) is too large to search exhaustively in efficient computational time, we developed a genetic searching optimization algorithm that searched over the entire domain of the 17 filtering parameters that characterize WGS data. This ‘genetic’ class of algorithm implements an objective function (‘fitness’) that is defined by several filters, and tries to minimize the value of the objective function over the entire domain (the lowest and highest possible values) of filters (every possible combination of filter choices) [17]. In our case we forced the sensitivity at various levels (0-100% in increments of 0.5%) and searched for the maximum specificity at each of those levels. Our objective function was a simple linear combination of all possible quality metrics for SNV data generated by GATK [1] and Picard (http://picard.sourceforge.net), with a varying polynomial coefficient matrix.

**Table 2:** Optimized filtering parameters applied by Platypus

**Filtering parameter**	**Optimized value**
**Filters tested and found to affect specificity and sensitivity**	
Alignment aggregate mapping quality	<7
Total quality	<196.5
Depth of coverage	<14
Strand bias Fisher’s exact test	>13.5
**Filters tested and found not to affect specificity and sensitivity**	
Count of nucleotide identity	n/a
Clipped read significance	n/a
Depth of coverage per allele	n/a
Quality by depth	n/a
Homopolymer run	n/a
Inbreeding coefficient	n/a
Allele balance	n/a
Confidence of elimination of incorrect genotype	n/a
Root mean square of mapping quality	n/a
Read position	n/a
Spanning deletions	n/a
Reads with mapping quality of zero	n/a
Reads with a mapping quality of zero	n/a

The final set of optimized filtering parameters evaluated by the searching algorithm. The set of informative filters gives 90% sensitivity with a specificity of 86%.

We chose a population of 10,000 parameter combinations to run through 100 evolutionary iterations. The algorithm we implemented included a low crossover rate (0.5) and high mutation rate (0.1) as well as a tournament pattern parental determination strategy with a tournament size of 100, and with a guaranteed 10 elite children using the MatLab Global Optimization toolbox. These settings were dynamically determined to give consistency and robustness across a variety of sensitivity ranges. Iterating through a forced sensitivity level in 1% increments yielded a smooth progression along a similar combination of filtering parameters.

The list of 10,000 randomly chosen parameter combinations was assessed for both sensitivity and specificity. Each set of filtering parameters sorted the true positives into two categories (“called” or “not called”) and similarly sorted the false positives; these calls were then evaluated for accuracy. Filtering sets that provided high specificity for a given level of sensitivity were carried over to the next round. The filtering parameters were then varied slightly within all successful sets, and individual parameters swapped between sets. After 100 iterative cycles, the most successful sets of filters converged on a single result – a theoretical optimal filtering set. We then added a further set of criteria based on the quality of the sequencing reads. The final optimized set excluded all SNV calls that met any of the following criteria listed in Table 2.

**Figure 1:**
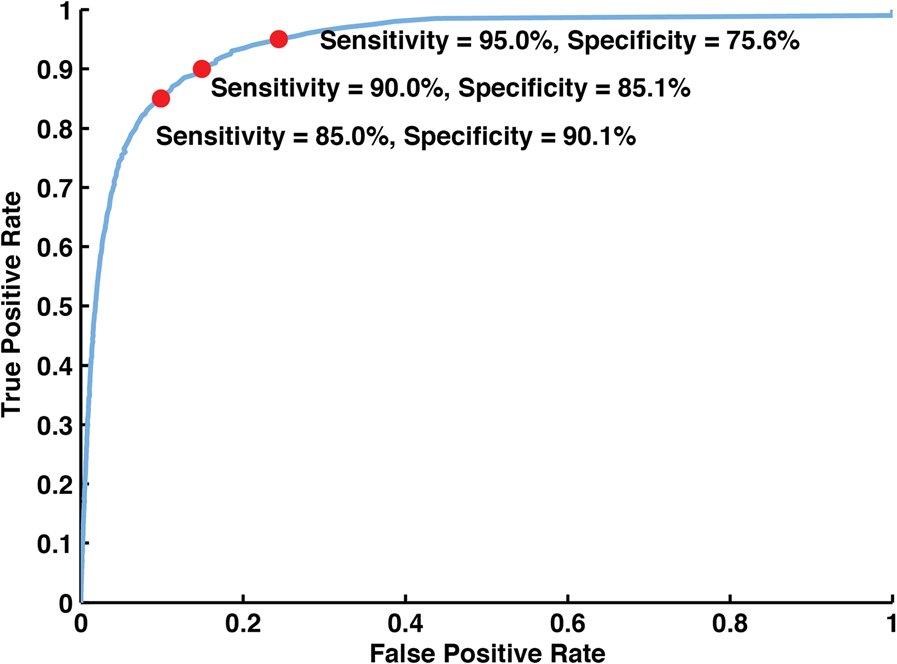
**Receiver operating characteristic for SNV optimization.** An ROC for the filtering parameters used in SNV detection optimization. Red points indicate the calculated specificity for certain fixed sensitivity levels. The blue line indicates the calculated specificity and sensitivity in 0.5% increments along the entire range of parameters. The optimal set of parameters for the GATK is given in Table 2. SNVs were then divided into either a ‘called or ‘not called’ category based on these metrics and the current set of filters being used. The following metrics were obtained from the Unified Genotyper: read depth, strand bias, haplotype score, homopolymer run length, total positional mapping quality, number of null mapping qualities, total positional quality, total quality by depth, and genotype quality (DP, FS, HaplotypeScore, HRun, MQ, MQ0, QUAL, QD, GQ). These SNVs were then hard filtered using the Variant Filtration Walker according to the given set of parameters, as in Table 2.

Using the optimal filtering set we detected 95.0% of the known SNVs with a specificity of 75.6% (8,315 total novel calls), and by lowering the total quality threshold we obtained a sensitivity of 90% of known SNVs, with a specificity of 85.1% (5,077 total novel calls). We generated a receiver operating characteristic curve (Figure 1) using optimal parameter sets at each sensitivity level, labeling three sensitivity threshold points of interest. Principal component analysis did not yield any statistically significant common genomic features (e.g. position on chromosome, position within gene, base pair transition) of the false positives detected, but 55% (4,598 calls) were found in the 12% of the genome we define as sub-telomeric (within 100,000 bp of the chromosomal end) and which likely lie in regions that were not adequately covered by the 7X Dd2 Sanger sequencing [14]. We also searched over a space of multi-dimensional filters (those depending on more than one quality metric in a nonlinear relationship). This included all multiplicative combinations of two and three parameters, as well as exponential, power, logarithmic, and quadratic functions of single parameters, but these filters were unable to find results that were as good as a combined one-dimensional approach (that is, better than the optimal specificity and any sensitivity level), possibly due to the computational complexity of searching over multi-dimensional filters. This set of filtering parameters (Table 2) is thus implemented in our pipeline and was used for all subsequent analyses.

CNVs contribute substantially to drug resistance in *Plasmodium* and other eukaryotic pathogens [18-21]. The current methods for calling CNVs in *Plasmodium* spp. WGS data, like most pathogenic eukaryotes, rely on smoothing the depth of coverage data (e.g. number of reads aligned to the reference) [22-24]. Smoothing is needed because sequencing depends on multiple stochastic processes and there can be great variability in the actual coverage over a given stretch of genomic DNA. Users are thus required to guess the appropriate smoothing parameters such as the number of base pairs to be averaged, meaning that the user already needs to know the approximate size of the CNV. Furthermore, it is known that there is also a non-stochastic bias in the depth of coverage due to the tendency of areas of high and low GC content to be sequenced less efficiently and this must also be accounted for, especially as *P. falciparum* is extremely AT-rich (81%). Because we found that the current algorithms produced a large number of false calls when applied to our WGS data, we sought to address this problem by developing our own CNV calling algorithm.

**Figure 2:**
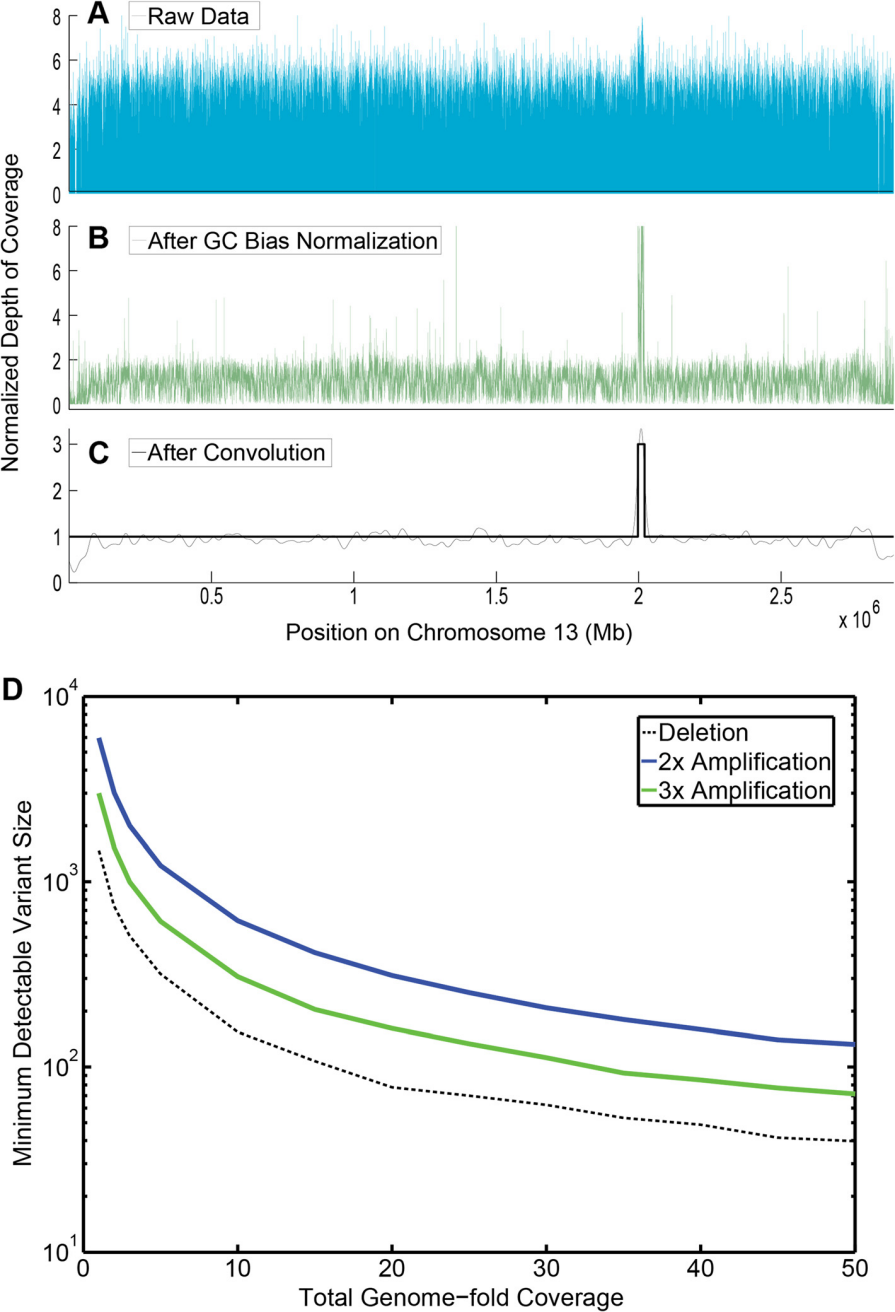
**CNV detection in a cladosporin resistant Dd2 line. A**. Raw depth of coverage data for chromosome 13 from the CladoR_clone3, which contains a microarray-verified amplification conferring resistance to cladosporin [10]. **B**. Depth of coverage normalized after GC bias correction. **C**. Normalized depth of coverage after the Platypus smoothing and edge-calling algorithm, with the actual copy number prediction overlaid. **D**. Theoretical minimum detectable variant size for a range of total genome coverage. Deletions are easier to detect and can be identified with less total coverage and in smaller sizes. Similarly, 3x amplifications are easier to detect than 2x.

The first improvement we made to the standard method was to improve GC bias correction. GC-content bias describes the dependence between fragment count (read coverage) and GC content found in high-throughput sequencing assays, particularly the Illumina Genome Analyzer technology. This bias can dominate the signal of interest for analyses that focus on measuring fragment abundance within a genome, such as copy number estimation and is not consistent between samples. We therefore analyzed regularities in the GC-bias patterns, and generated a closed-form compact description for this curve family. It is the GC content of the full DNA fragment (generally 100-200 bp), not only the sequenced read (the 50-100 bp sequenced on each end), that most influences this bias [9]. This GC bias distribution is unimodal: both GC rich fragments and AT rich fragments are under-represented in the sequencing results. Based on these findings, we devised a new method to calculate predicted coverage and correct for the bias.In order to implement the correction we first calculate the GC content of the sequenced fragment (for paired end reads, both reads plus the insert) after aligning the reads to the reference genome (PlasmoDB 7.1 for imidazolopiperazines, 10.1 for the remainder), assuming that the insert matches the reference genome between the reads exactly. While this assumption does not account for potential point mutations, we have tested the effect of this on the bias normalization algorithm, and it is imperceptible, because point mutations occur so infrequently in a long insert size that the total GC content is not affected. After GC normalization CNVs become much more apparent than they were in the raw data (compare Figure 2A and B).

Secondly, after GC content normalization, the depth of coverage must be ‘smoothed’ so that true CNVs can be detected and random fluctuations in the data can be identified and discarded. However, smoothing algorithms tend to blur the divisions between otherwise sharp edges. Thus we also developed a simple iterative process of smoothing and edge detection that would identify these boundaries. This step integrates an iterative Weierstrass transform followed by edge detection through convolution with a first-derivative Gaussian kernel [25]. These can be described as follows: Let *D*_*n*_ be the smoothed depth of coverage of a chromosome of length *l* normalized after *n* iterations. Then, with a Gaussian kernel *G* with arbitrary coefficient *k*, we have, for all positions *j* on the chromosome, *χ*_*j*_:

$$D_{n}=G*D_{n-1}$$

Which we can expand to:

$$D_{n}\left(\overset{\to }{x}\right)=\overset{l}{\underset{i=1}{\sum }}\left(k_{1}e^{-k_{2}\left(\overset{\to }{x}-i\right)∙\left(\overset{\to }{x}-i\right)}D_{n-1}\left(i\right)\right)$$

We can detect the edges of this data by finding all solutions:

$$x\in l:\frac{\partial }{\partial x_{j}}\left(\frac{\partial G}{\partial x_{j}}*D_{n}\right)=0$$

Which is expanded to:

$$x\in l:\overset{l}{\underset{i=1}{\sum }}\left(-2k_{1}k_{2}e^{-k_{2}\left(\overset{\to }{x}-i\right)∙\left(\overset{\to }{x}-i\right)}D_{n}\left(i\right)+4k_{1}k_{2}^{2}{\left(x-i\right)}^{2}e^{-k_{2}\left(\overset{\to }{x}-i\right)∙\left(\overset{\to }{x}-i\right)}D_{n}\left(i\right)\right)=0$$

An example of the output of this algorithm is demonstrated in Figure 2C.

To save computational time, we applied the convolution theorem to take these operators in the Fourier space and as such, reduce all operations to point-wise multiplication. After each Weierstrass transform, edges are detected by the above formula. The total number of convolution iterations was set to be variable in the first *in silico* tests, ending only when no new edges had appeared in the last 10 iterations, but was eventually held constant at 5 because in practice no new edges appeared after the 2nd or 3rd iterations of the algorithm. We must treat the mitochondrial and apicoplast genome separately, as the depth of coverage of these is usually very different than the other *Plasmodium* chromosomes, even by an order of magnitude. The depth of coverage in each region (i.e. between each edge) is then compared to the sample mean, and those that are statistically higher or lower are assigned an amplification number based on their increase (or decrease) relative to the mean.

Recombination contributes substantially to the virulence of many eukaryotic pathogens such as *P. falciparum* and *T. bruceii* where genome encoded virulence factors are located in hyper-recombinogenic sections of the genome. In addition, such rearrangements could contribute to a phenotype if no causative SNV or clear dosage effect in a likely target is found. We thus sought to implement a program to find these recombination events.

**Figure 3:**
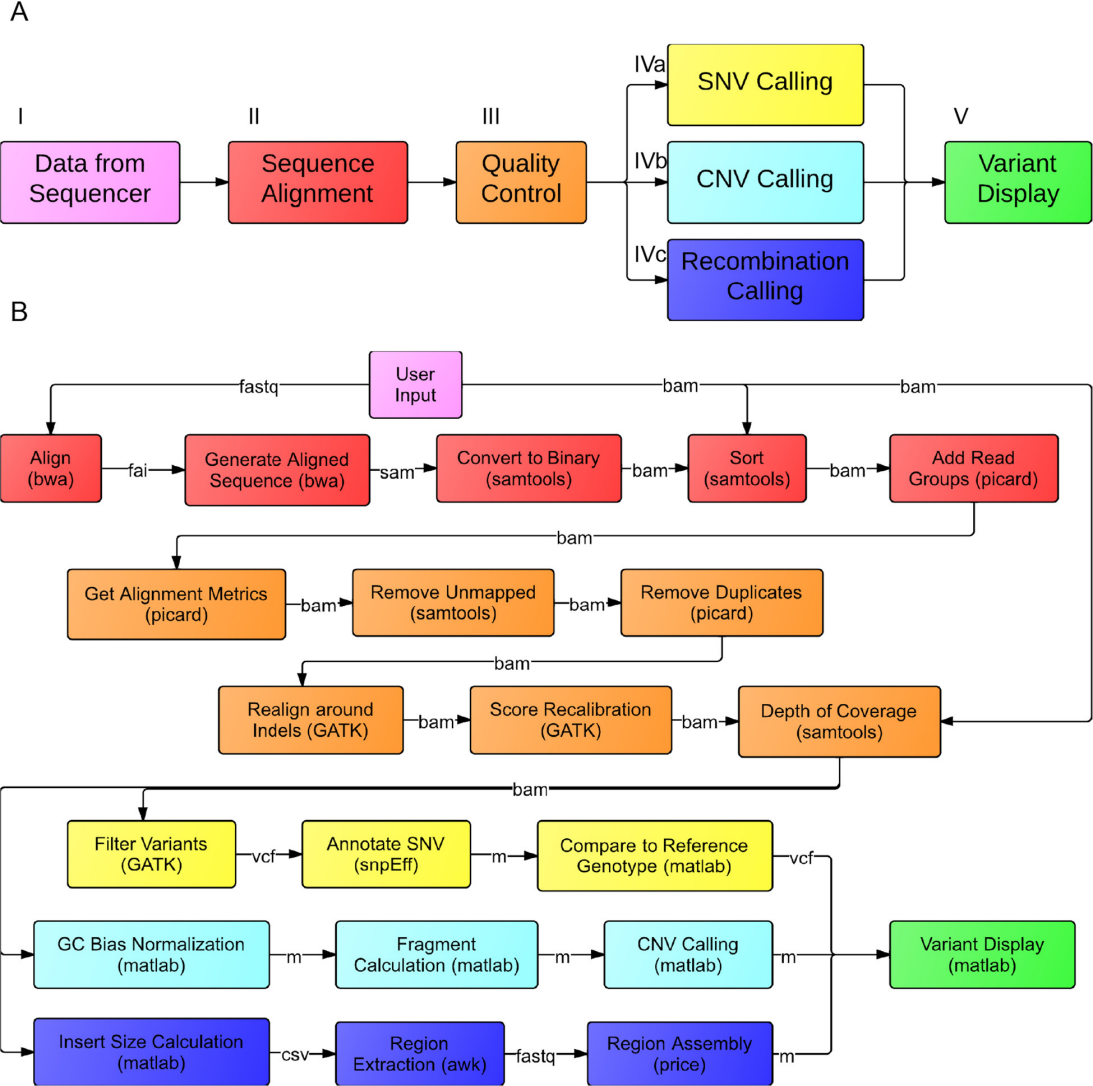
**Schematic of the Platypus data processing pipeline.** Diagram of all the external and internally developed programs involved in processing *Plasmodium* samples as implemented by the Platypus pipeline. The SNV analysis is coded as a sequence of C programs, combining programs written by a variety of other research groups in Java and C. These programs used are the Burrows-Wheeler Aligner [27], Sequence Alignment Map Tools [13], the Genome Analysis Toolkit [1], and the Picard tool set. Each script encodes a single step of the data cleaning and analysis pipeline, including error checking and customization of the program. The GC bias normalization algorithms and the CNV detection algorithms were written in MATLAB (The Mathworks) and coded in C after export, and are integrated into the pipeline as shell scripts as well. Reads are aligned to the *P. falciparum* 3D7 reference genome version using the Burrow-Wheelers Aligner. Alignment files are then converted to a binary map and sorted and indexed using SAMTools. (steps I-II, A) Sequencing run statistics for each sample, including GC bias metrics and quality score distribution statistics, are then collected using a number of Picard programs. Read group identifiers are then added, and unmapped reads are removed from the alignment using SAMTools. Next optical and PCR duplicates are removed using Picard, and the entire alignment is realigned around possible insertion-deletion events using GATK. Base quality scores are recalibrated using GATK, and the depth of coverage at every base pair position is calculated. (step III, A) The alignment is then indexed and is ready for normalization and analysis. **A**. Schematic of entire workflow, with color-coding corresponding to the steps in **B**. Diagram of every program and action used in the Platypus pipeline, with file types traced passing between programs written in between. We request that all users of the Platypus acknowledge both this manuscript and the referenced manuscripts for the other programs included in the manual. Please see the instruction manual, available on the website, for more information.

Our strategy was to identify fragments with mated pairs that had abnormal insert sizes when they were aligned to a reference genome, especially ones with mated pairs that aligned to two different chromosomes or to vastly distant parts of the same chromosome. To find the initial events, all reads which had a mates aligning farther than 10 kb away from each other or on another chromosome were extracted and each region with a group of 10 or more overlapping reads with this property was *de novo* assembled using PRICE [26] with 20 cycles and otherwise standard settings (-icf $x 1 1 5, -nc 20, -dbmax 100, -maxHp 7, -lenf 2500 20). These contigs were then aligned against the reference genome using ClustalW to discover the origin of each part of the contig. Figure 3A, step IVc outlines the recombination detection process.

## Results and discussion

To further test the validity of our optimized SNV filtration, and to test the accuracy of the predicted sensitivity (90%) and specificity (85%), we next evaluated the algorithm’s ability to find rare mutations in isogenic lines created in our laboratory. We gathered WGS data from five separate chemical resistance experiments involving known and experimental antimalarial compounds (Table 1). In these experiments a clonal 3D7 or Dd2 line was exposed to sub-lethal concentrations of a small molecule inhibitor for 2–4 months. Parasites were then sub-cloned by limiting dilution and genomic DNA (gDNA) was sequenced, along with the gDNA of their isogenic parental clone. All strain genotype data were compared to the parent so that only genomic changes arising after chemical selection pressure would be identified. These strains have demonstrated resistances to a variety of small molecule inhibitors (atovaquone [6], cladosporin [10], spiroindolones [11] and imidazolopiperazines [12]). Most of these strains were initially characterized using a custom Affymetrix *P. falciparum* tiling microarray [28] and confirmed using Sanger sequencing or qPCR but some had been whole-genome sequenced previously, and some were sequenced specifically for this study (cladosporin resistant line). Altogether we analyzed data from 26 clones with their respective 2 parents. All were sequenced to 25-83X coverage using paired-end reads with ~150 bp fragment size on the Illumina Genome Analyzer II and Illumina HiSeq platforms (Tables 1 and 3).

**Table 3:** Total number of SNVs detected using Platypus compared to simple filtering

**Experiment ID**	**# Raw SNVs**	**# SNVs from Q30**	**# SNVs from Platypus**	**Amino acid change conferring resistance**	**Genome total-fold coverage**
KAD707B1	613972	20	12	L830V, M1069I	43x
KAD707B2	50145	10	6	M81I, L830V	62x
KAD707B3	52389	5	3	L830V, S1076I	50x
KAD458	60250	13	8	S1076I, V1053A	25x
CladoR_clone1	82488	53	32	n/a (CNV)	67x
CladoR_clone2	100033	80	48	n/a (CNV)	42x
CladoR_clone3	107611	68	41	n/a (CNV)	47x
NITD609_1	50008	8	5	I398F, P990R, CNV	49x
NITD609_2	48201	12	7	T418N, P990R	48x
NITD609_3	48484	13	8	D1247Y	36x
NITD678_1	51665	18	11	G223R	39x
NITD678_2	56756	15	9	A184S, P990R	42x
NITD678_3	83190	55	33	I203M, I263V	44x
EvoR1a	45388	8	5	M133V	83x
EvoR1a2	46151	12	7	M133V	76x
EvoR1b	46462	10	6	M133V	60x
EvoR1b2	46377	15	9	M133V	79x
EvoR2a	45565	3	2	M133I	64x
EvoR2a2	45604	3	2	M133I	67x
EvoR2b*	n/a*	n/a*	n/a*	n/a*	1x
EvoR2b2	38188	3	2	M133I	82x
EvoR3a	38224	7	4	M133I	54x
EvoR3b	38620	7	4	M133I	62x
EvoR4a	37404	5	3	M133I, L144S	67x
EvoR4b	38333	5	3	M133I, L144S	78x
EvoR5a	46563	3	2	F267V	79x
EvoR5b	46890	3	2	F267V	65x

*Sequencing data dominated by contamination.

Number of raw SNVs as well as the number of called SNVs from the Q30 metric [29] and the Platypus’ final output. The Dd2 and 3D7 parents were compared to the 3D7 reference sequence from PlasmoDB v9.1, and then the drug resistant samples were compared to their respective parent. Results were compared to SNVs found by microarray or Sanger sequencing to assess sensitivity and specificity.

**Figure 4:**
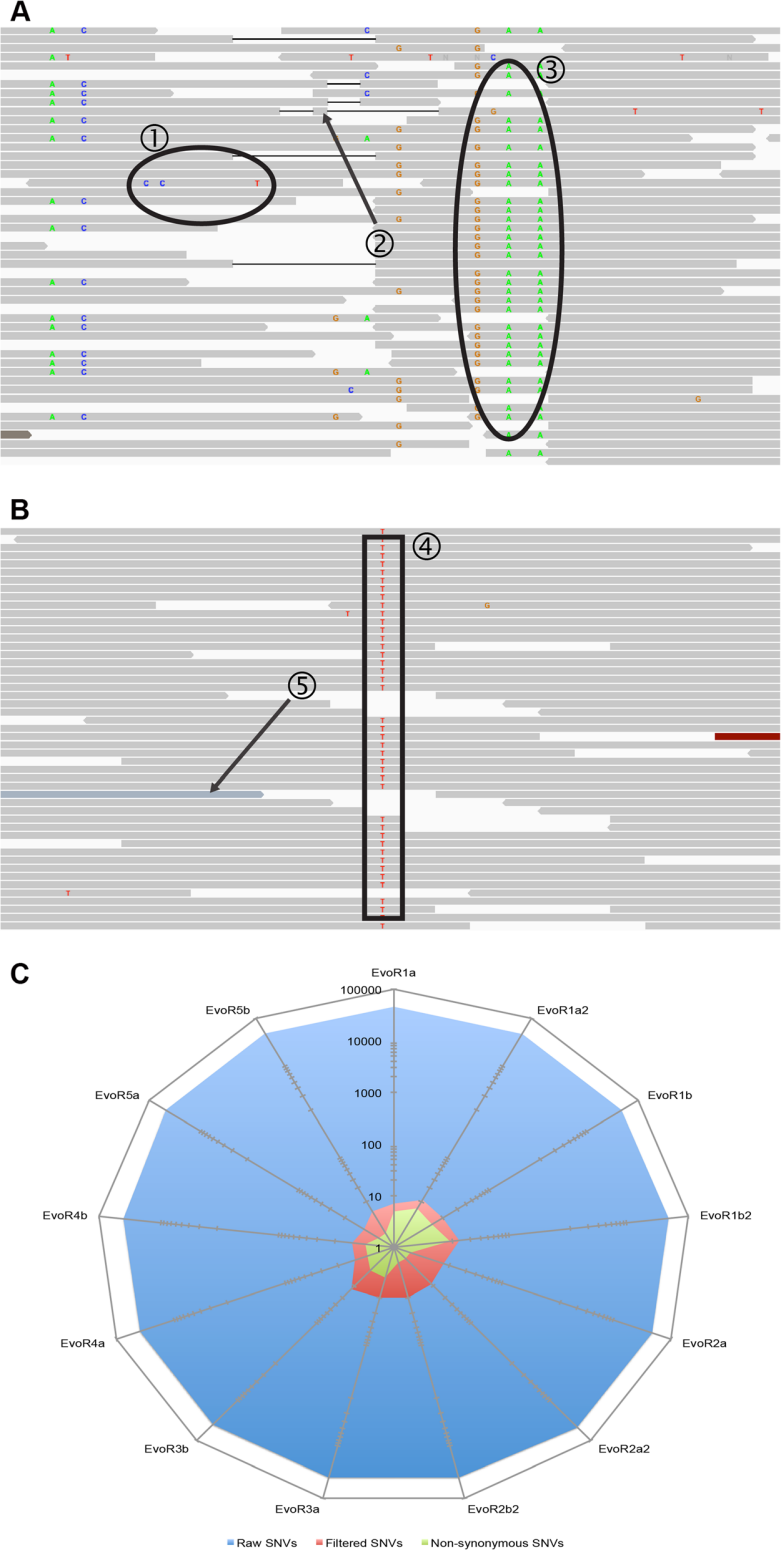
**SNV filtering diagram for the Evo Samples. A**. Read pileup of a region from EvoR1a2 including miscalled nucleotides within a read (1), misaligned reads (2 and 3), and false SNV calls due to a homopolymer run followed by a repetitive region. This region is an example of the false positive that the Platypus seeks to eliminate. **B**. Read pileup of a region from EvoR1a2 including a true SNV (4) but still including a number of misaligned reads (5). The Platypus seeks to extract these true positive SNVs from their surroundings. **C**. Shows the number of raw SNV calls (blue) and the total number after filtration (red) and the subset of nonsynonymous SNVs (green) that may cause a change in the drug efficacy profile. Scale is logarithmic – the Platypus produces a 10^3^-10^4^x reduction in SNV calls.

For each of the 26 whole genome sequencing sample datasets, the Platypus pipeline was able to analyze and filter the sequencing data from tens of thousands of SNVs (examples of which are shown in Figure 4A) to a median of 6 final SNV calls (Figure 4B,C). The total number of raw SNVs, the number after filtering, and the number of these that were nonsynonymous for each of the experiments, are given in Table 3. These results included 100% sensitivity concordance with the respective microarray and sequencing results, including all of the 20 unique SNVs that had been previously implicated in drug resistance (Table 3) as well as 127 additional mutations. More importantly, Platypus detected all SNVs that conferred the respective drug resistance phenotype and highlighted those which would cause a nonsynonymous change in an amino acid and alter protein function. Altogether, 63 SNVs in the atovaquone, spiroindolone, and cladosporin resistant samples that were output by the Platypus program were confirmed as true hits by Sanger sequencing. In addition, 52 SNVs in the atovaquone resistant samples that were closest to the cutoff line, but still excluded, were chosen as validation targets of a true negative result. These regions were sequenced and found to be insertion/deletion events, which our program is not currently designed to detect. These results confirm that the program is robust and does not misinterpret sequencing data for *Plasmodium* spp.. In addition, the power of the program is clear – in each sample the Platypus filtered out everything (to the best of our knowledge) except that which was most relevant to the experiment. The time and effort saved by removing the long process of manual curation will have a major impact on the analysis of WGS data for *Plasmodium* and eukaryotic pathogen genomes in general. These methods can be directly applied to a variety of other pathogens as well, and we hope to see collaborative efforts to expand the pipeline to other organisms.

We note that the Platypus reduces the total number of SNVs from raw data by a factor of approximately 10^3^-10^4^ (Table 3). While we cannot comprehensively genotype the tens of thousands of SNVs called initially by GATK or SAMTools, we have verified in atovaquone, spiroindolone, and cladosporin resistant lines that 63 of the SNVs called by Platypus are true genetic variants, and none of the 52 sites from the atovaquone resistant samples were excluded erroneously.

We also see (Table 3) that a comparison to the Q30 metric [29] identifies the Platypus as having significant gains over this simpler metric, reducing the number of SNVs called by a factor of approximately 1.6x. The sites called by the Q30 metric and excluded by the Platypus constituted 48 of the 52 sites that were Sanger sequenced and subsequently discovered to be not true, validating their exclusion by the Platypus.

There is no standard set of filtering parameters to use with GATK, but we can compare to a set of published filter values for a comparable project [30]. Using Bright et al. as a comparison point, we can adapt their filters into our current pipeline. Doing so yields a 91% sensitivity level with a specificity of 45%. We can see that these heuristically chosen values have a reasonable sensitivity threshold but do not hold up to empirically designed filters in terms of specificity.

The assessment of a false positive and false negative rate can of course never be perfected, but in all cases we have detected plausible drug resistance genes in all cases. Comparison with known values and with extensive Sanger sequencing data confirms our calls, and even indicates that these sets of filters may be too lenient – that we may be detecting nonexistent SNVs rather than missing true ones.

To determine the sensitivity and specificity of the CNV detection algorithm, we sought to detect eight known amplifications from WGS data of the 26 strains with known structural variants, all of which had been discovered using our custom tiling microarray (Table 4). Figure 2A shows the raw depth of coverage data for a known amplification conferring cladosporin resistance on chromosome 13, and also indicates both the depth of coverage data after applying the detection algorithm (Figure 2B) and shows the copy number variant called from the data (Figure 2C).

**Table 4:** CNVs detected using WGS and genomic microarrays

**Strain**	**Position (Micr.)**	**Position (Seq.)**	**Presumed relevant gene**	**Copy number (Seq.)**	**Orientation (Seq.)**
all 3D7 derived lines	chr12_974243-975980	chr12_974276-976007	*pfgch*	2	tandem
all Dd2 derived lines	chr5_892863-968421	chr5_892872-968429	*pfmdr1*	2	tandem
all Dd2 derived lines	chr12_970985-975864	chr12_971011-975866	*pfgch*	2	tandem
Evo5a/b	chr1_428540-643352	chr1_428538-643350	*pfmrp1*	2	unknown*
CladoR_clone1	chr13_1996541-2018534	chr13_1996635-2018727	*pfkrs1*	3	tandem
CladoR_clone2	chr13_1996668-2055132	chr13_1996621-2055107	*pfkrs1*	3	tandem
CladoR_clone3	chr13_2001466-2051201	chr13_2001482-2051233	*pfkrs1*	3	tandem
NITD678_clone1	chr12_510069-633784	chr12_510123-633834	*pfatp4*	2-3	tandem

*Orientation could not be determined as the amplification continued into the telomeric region.

Predicted CNV calls with position from both WGS (Seq.) using Platypus and microarray data (Micr.), with predicted copy number compared to the 3D7 reference and orientation of the amplification as determined by Platypus.

Altogether Platypus identified all 8 unique CNVs that were known to exist in our strains. Our algorithm identified the large ~100 kb CNV surrounding the *P. falciparum* multidrug resistance protein-1 gene (*pfmdr1*, PF3D7_0523000) in the 13 Dd2 derived strains [20,24] and the 5 kb GTP cyclohydrolase amplifications in 13 Dd2 (*pfgch*, PF3D7_1224000) derived strains as well as the smaller 1.6 kb amplification GTP cyclohydrolase in 13 3D7 derived strains [19]. We were also able to identify several independent larger amplifications that included lysyl tRNA synthetase (*pfkrs1*, PF3D7_1350100) in 3 strains that are resistant to cladosporin (a drug which targets lysyl-tRNA synthetase) [10], and an amplification on chromosome 1 in the EvoR5 strain that was grown in the presence of atovaquone, both confirmed by microarray as well [6]. We were also able to detect an amplification on chromosome 12 (containing *pfatp4*) in 3 of the spiroindolone resistant samples [6,11]. Although there was some ambiguity as to the number of copies (i.e. duplication or triplication), the Platypus also reported a SNV in one copy of *pfatp4* but not in the other copies of the gene. Furthermore, we discovered no spurious or novel amplification or deletion events, i.e. CNVs that were not detected by tiling microarray.

**Figure 5:**
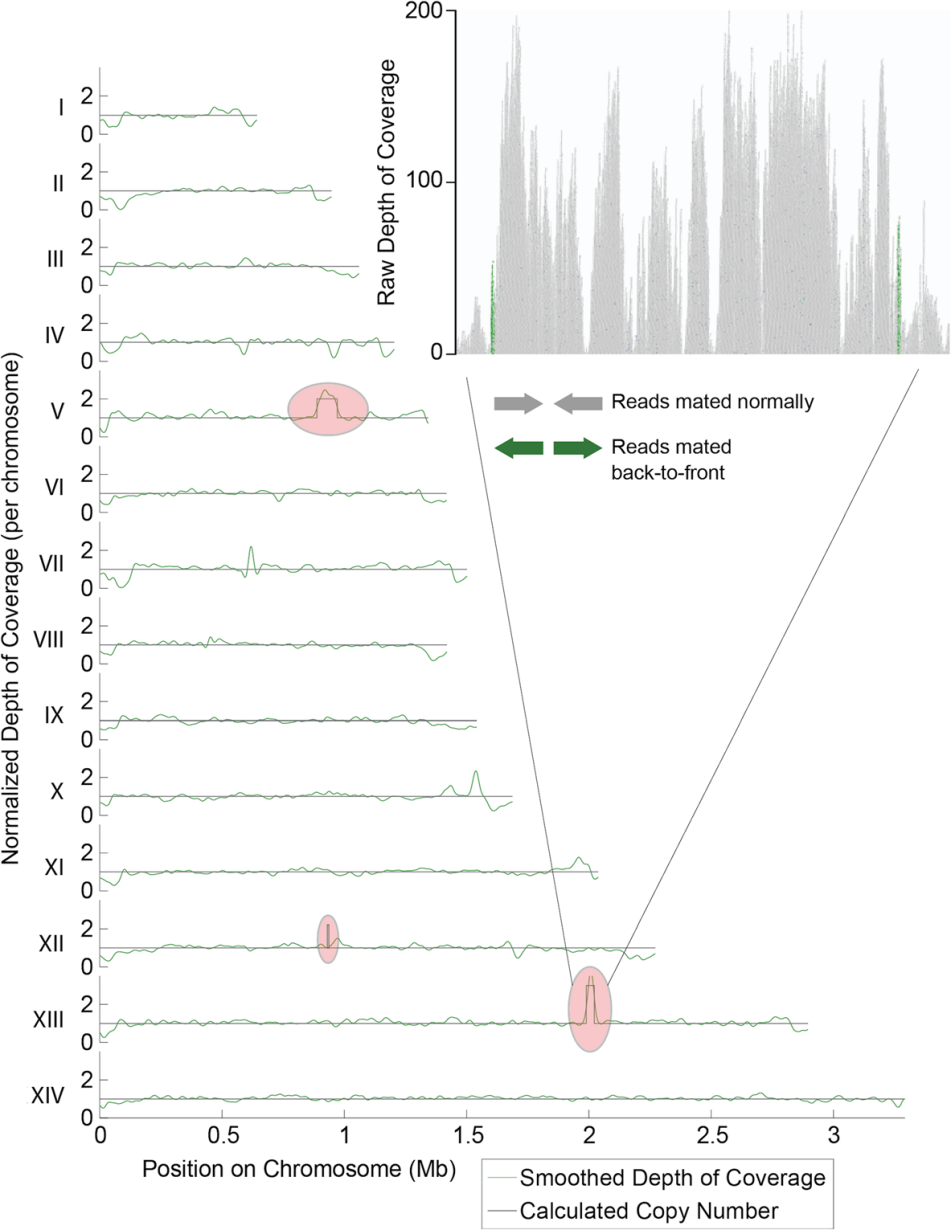
**Detecting genome amplification events.** Depth of coverage data for all 14 chromosomes of a Dd2 clonal line with evolved resistance to cladosporin (CladoR_clone3) [10]. The curve represents the output of the CNV detection algorithm, while the black line is the actual copy number call by the program. The two visible amplifications detected are the one encompassing the *pfmdr1* locus on chromosome 5 and the amplification associated with cladosporin-resistance on chromosome 13 [10]. A third amplification on chromosome 12 containing *pfgch* is visible at higher magnification. The inset shows a read pileup for the region containing an amplification on chromosome 13. The green colored reads indicate those with an abnormal insert size with mated reads mapping back-to-back rather than front-to-front. This is evidence that the amplification is in tandem with the original copy on the chromosome.

In addition, the boundaries for the respective CNV called by the algorithm for each sample correspond closely to the boundaries detected by microarray – the edges of the CNV algorithm lie, almost exclusively, within 100 bp of the probes near the amplification boundaries. To further validate these data we also examined the read pileup near the predicted boundaries (Figure 5) for some cases (CladoR_clone1 shown). The read pileup allowed us to investigate the orientation of the amplification event. In the Dd2 lines mentioned above (those with detected amplifications surrounding *pfmdr1*, *pfgch*, and *pfkrs1, pfatp4*), 46-58% of the paired-end reads at the beginning of each amplification mated to the end of that amplification with an abnormally large insert size and in the reverse read orientation (Figure 5, inset). This indicates that the amplification event is not only on the same chromosome but also in tandem – that is, the amplified genomic region is adjacent to the originating sequence (Table 4). Interestingly, we found all amplifications that we examined were in a tandem orientation.To test the theoretical limits of the CNV detection algorithm we also generated simulated amplifications and deletions by producing a depth of coverage from a random distribution that had a mean twice the sample mean (amplifications), or by completely eliminating certain reads (deletions). Deletions could be detected with low coverage and in very small sizes, with a theoretical limit around 3x coverage and 500 bp (with 100 bp reads). Amplifications were harder to detect, but the limit is approximately 1000 bp at 20x total genomic coverage. This limit is accurate for all deletions and for amplifications up to four times, after which point the algorithm can only detect the presence of a variant and estimate its copy number (an estimate of 6x indicates a 5x-7x CNV). Figure 2D demonstrates the theoretical capabilities of the algorithm in detecting both deletion and amplification events for various total genome coverage levels.

We compared our CNV calling algorithm to BreakDancer, a similar program used to detect both copy number and recombination events using the default [31]. Using a set of parameters equivalent to those published in Chen et al., (4 standard deviation threshold, Q>39, MQ>35) we see that BreakDancer is fully able to detect all CNVs present in our samples (those detected by microarray and/or whole genome sequencing), but it also identifies 73 other CNVs ranging from 434 bp to 11639 bp that we do not detect by any method. Indeed, qPCR amplification of these speculated regions indicates no change in copy number in any of these regions not detected by other high throughout methods.

**Figure 6:**
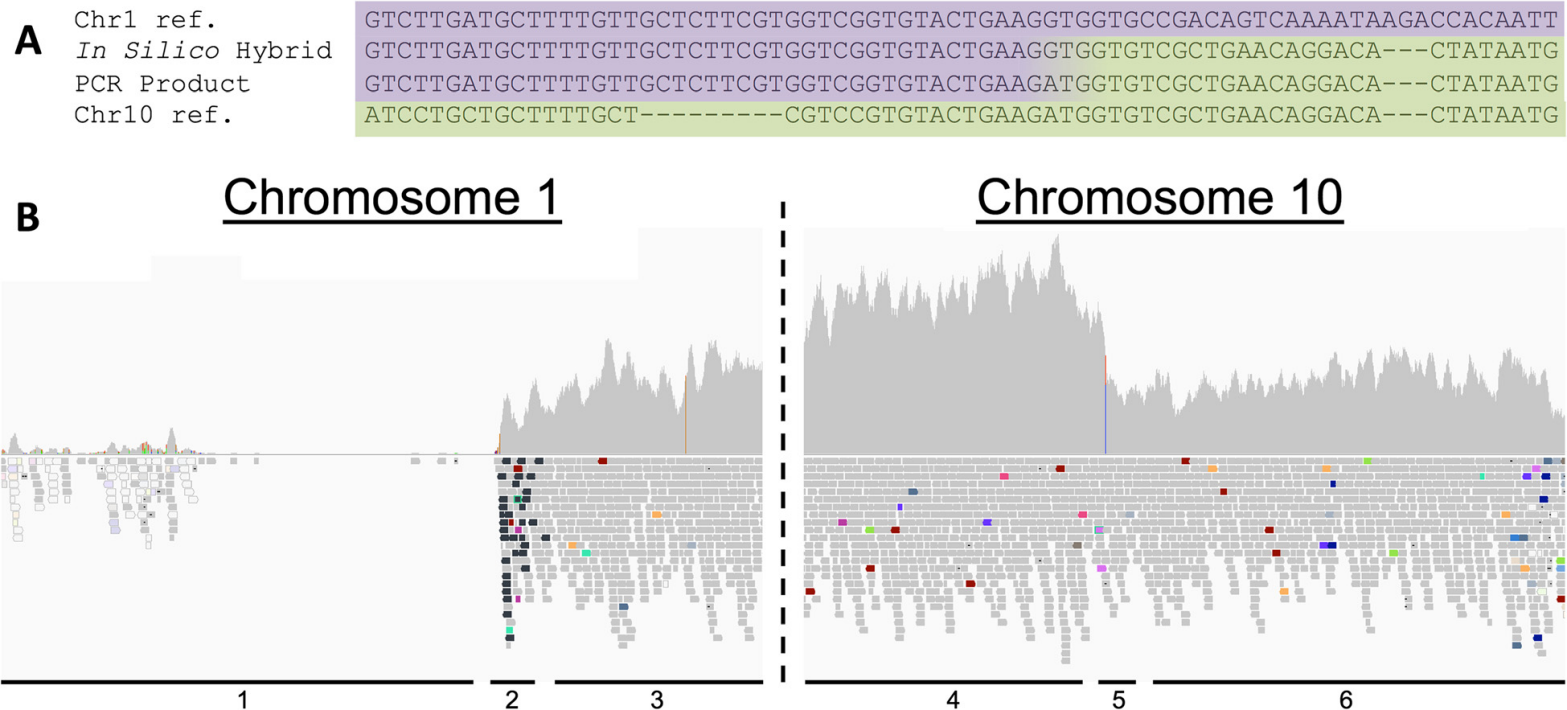
**Recombination events detected in long term culture samples. A**. Reassembled sequences from recombination events indicate a splicing of 56,000 base pairs from the left sub-telomeric region of chromosome 1 on to the right sub-telomeric region of chromosome 10. The center sequence is the assembled recombination from the sequencing data, while the top and bottom are the respective reference sequences for each of the indicated chromosomal regions. Note the clear transition between alignments. **B**. Read pileup for the two regions containing the predicted recombination. Note the deletion (region 1) on chromosome 1 next to the breakpoint (region 2) and subsequent normal coverage (region 3). Similarly, chromosome 10 shows an amplification event (region 4) next to the breakpoint (region 5) and then subsequent normal alignment (region 6).

Our algorithm identified 15 of these potential interchromosomal events in 13 3D7 Evo strains that resulted in partial gene deletion/duplication paired events, which also registered as deletion/amplification events in the CNV algorithm. In order to validate that these were indeed recombination events, the PRICE assembler was seeded with a single read from the suspected recombination region, and then *de novo* assembly was carried out using all reads that surrounded the alignment of the seeded read and its mate pair, as well as their respective mates. Altogether we were able to validate all 15 of these likely recombination events despite the hypervariability and high AT content (as high as 95% in these regions) that makes the specificity and uniqueness of these sequences very low. Figure 6 shows the sequence of one of the predicted regions as well as the matching sequence of the PCR product. To seek further evidence of genomic rearrangement, the 15 reassembled recombination events were validated by Sanger sequencing using primers that spanned the region. We found that the sequence of the *de novo* assembled fragment matched that of the Sanger sequencing product to within a one base pair difference in all 15 events. These assemblies spanned chromosomal sub-telomeric regions, and spliced together related genes. The PCR sequences of these assemblies and their clustal alignments are included in the supplemental information, and the mapping of each recombination event to each isolate is included in the supplement (see Additional file 1: Table S1). BLAST/CLUSTALOmega analysis verifies that these regions map to two separate chromosomes as expected.

## Conclusions

A problem with using WGS is that it may be inaccessible to laboratories that are not strong in bioinformatics. To address this issue we integrated these modules into a program that we call Platypus (Figure 3). The pipeline integrates a number of other software programs, and these are referenced in full in this manuscript and in the software documentation. Platypus takes as input either unaligned FastA/FastQ sequencing data, or aligned data in the BAM format. SNVs, CNVs, and potential recombination events are output as annotated text files which can be cross-referenced with PlasmoDB or similar databases.

The Platypus pipeline provides malaria researchers with a powerful tool to analyze short read sequencing data. It provides an accurate way to detect SNVs using known software packages, and a novel methodology for detection of CNVs, though it does not currently support detection of small indels. We have validated that the pipeline detects known SNVs in a variety of samples while filtering out spurious data. We have also tested it against both computational samples and actual data with known CNVs (both deletions and amplifications as verified by microarray) and it can detect the size and boundaries of these CNVs with a high degree of accuracy. The success of the Platypus software in both detecting real genetic variants and avoiding the reporting of false positives over a number of parasite lines can be attributed to its basis on first principles. The SNV detection was specifically designed only to use filters that accurately segregated true and false positives, and the robustness of this approach is evident, as there is a completely smooth transition between sensitivity/specificity levels when varying over the ideal filter set. The CNV detection was based on the fundamental theorem of digital signal processing, and indeed the assumptions of this field applies directly to the signals coming off a next-generation whole genome sequencer, complete with random and systematic biases. This streamlined package offers an initial starting point for the field to analyze and report these data in a consistent manner.

## Availability and requirements

The program is platform independent and can be run on ordinary desktop computers: In our case all analysis and computer programming was done using Mac OSX 10.7.3 on a Mac Pro with 12 multi-threaded processors on 2 cores and 32Gb of 1066 MHz DDR2 RAM. Altogether 24Gb of RAM was made available to Java while the Platypus was running. We have made Platypus freely available as an open-source package at <http://sourceforge.net/projects/platypusmga/>.

## Abbreviations

Bp: Base pair; CNV: Copy number variant; DNA: Deoxyribonucleic acid; FastA: Fast (all) format; FastQ: Fast (all) quality format; GATK: Genome analysis toolkit; gDNA: Genomic DNA; Mb: Mega- base pair; PlasmoDB: Plasmodium database; Platypus: Pathogen lovers automated type uncovering software; SAM: Sequence alignment map; SNV: Single nucleotide variant; *spp*: Species pluralis; WGS: Whole genome sequencing.

## Competing interests

The authors declare that they have no competing interests.

## Authors’ contributions

MJM designed the program, aligned the sequences, wrote the computer algorithms, ran the validations, performed the human analysis, and wrote the initial drafts. SSS performed human analysis and ran validation experiments. ELF provided extensive manuscript preparation and program testing. SERB cultured parasites and analyzed the data for the evolution experiment. VCC assisted in program validations and aligning sequences and assisted in manuscript preparation. ATB assisted in the program development and validation. CWM cultured parasites and assisted in the analysis of the cladosporin experiment. JRW sequenced all the samples and assisted in the initial analysis. EAW helped design the program, assisted in validation strategies, and assisted with writing the manuscript. All authors read, reviewed, critiqued, and approved the full manuscript.

## Supplementary Material

Additional file 1: Table S1 Presence (1) or absence (0) of potential recombination events in various isolates from the evolution experiments (Bopp et al, 2013) [6]. Click here for file

## References

[B1] McKenna A Hanna M Banks E Sivachenko A Cibulskis K Kernytsky A Garimella K Altshuler D Gabriel S Daly M DePristo MA The Genome analysis toolkit: a MapReduce framework for analyzing next-generation DNA sequencing data Genome Res 2010 20 1297 1303 10.1101/gr.107524.110 20644199 PMC2928508

[B2] Gardner MJ Hall N Fung E White O Berriman M Hyman RW Carlton JM Pain A Nelson KE Bowman S Genome sequence of the human malaria parasite Plasmodium falciparum Nature 2002 419 498 511 10.1038/nature01097 12368864 PMC3836256

[B3] Manske M Miotto O Campino S Auburn S Almagro-Garcia J Maslen G O’Brien J Djimde A Doumbo O Zongo I Analysis of Plasmodium falciparum diversity in natural infections by deep sequencing Nature 2012 487 7407 375 379 10.1038/nature11174 22722859 PMC3738909

[B4] Miotto O Almagro-Garcia J Manske M Macinnis B Campino S Rockett KA Amaratunga C Lim P Suon S Sreng S Multiple populations of artemisinin-resistant Plasmodium falciparum in Cambodia Nat Genet 2013 45 6 648 655 10.1038/ng.2624 23624527 PMC3807790

[B5] Samarakoon U Regier A Tan A Desany BA Collins B Tan JC Emrich SJ Ferdig MT High-throughput 454 resequencing for allele discovery and recombination mapping in Plasmodium falciparum BMC Genomics 2011 12 116 10.1186/1471-2164-12-116 21324207 PMC3055840

[B6] Bopp SER Manary MJ Bright AT Johnston GL Dharia NV Luna FL McCormack S Plouffe D McNamara CW Walker JR Mitotic evolution of Plasmodium falciparum shows a stable core Genome but recombination in antigen families PLoS Genet 2013 9 e1003293 10.1371/journal.pgen.1003293 23408914 PMC3567157

[B7] Murray CJ Rosenfeld LC Lim SS Andrews KG Foreman KJ Haring D Fullman N Naghavi M Lozano R Lopez AD Global malaria mortality between 1980 and 2010: a systematic analysis Lancet 2012 379 413 431 10.1016/S0140-6736(12)60034-8 22305225

[B8] Trager W Jensen JB Human malaria parasites in continuous culture Science 1976 193 673 675 10.1126/science.781840 781840

[B9] Benjamini Y Speed T Estimation and correction for GC-content bias in high throughput sequencing 2011 Berkeley, CA USA: Tech Rep 10.1093/nar/gks001 PMC3378858 22323520

[B10] Hoepfner D McNamara CW Lim CS Studer C Riedl R Aust T McCormack SL Plouffe DM Meister S Schuierer S Selective and specific inhibition of the plasmodium falciparum lysyl-tRNA synthetase by the fungal secondary metabolite cladosporin Cell Host Microbe 2012 11 654 663 10.1016/j.chom.2012.04.015 22704625 PMC3391680

[B11] Rottmann M McNamara C Yeung BK Lee MC Zou B Russell B Seitz P Plouffe DM Dharia NV Tan J Spiroindolones, a potent compound class for the treatment of malaria Science 2010 329 1175 1180 10.1126/science.1193225 20813948 PMC3050001

[B12] Meister S Plouffe DM Kuhen KL Bonamy GM Wu T Barnes SW Bopp SE Borboa R Bright AT Che J Imaging of Plasmodium liver stages to drive next-generation antimalarial drug discovery Science 2011 334 1372 1377 10.1126/science.1211936 22096101 PMC3473092

[B13] Li H Handsaker B Wysoker A Fennell T Ruan J Homer N Marth G Abecasis G Durbin R Subgroup GPDP The sequence alignment/map format and SAMtools Bioinformatics 2009 25 2078 2079 10.1093/bioinformatics/btp352 19505943 PMC2723002

[B14] Volkman SK Sabeti PC DeCaprio D Neafsey DE Schaffner SF Milner DA Jr Daily JP Sarr O Ndiaye D Ndir O A genome-wide map of diversity in Plasmodium falciparum Nat Genet 2007 39 113 119 10.1038/ng1930 17159979

[B15] Aurrecoechea C Brestelli J Brunk BP Dommer J Fischer S Gajria B Gao X Gingle A Grant G Harb OS PlasmoDB: a functional genomic database for malaria parasites Nucleic Acids Res 2009 37 D539 D543 10.1093/nar/gkn814 18957442 PMC2686598

[B16] Barry AE Leliwa-Sytek A Tavul L Imrie H Migot-Nabias F Brown SM McVean GA Day KP Population genomics of the immune evasion (var) genes of Plasmodium falciparum PLoS Pathog 2007 3 e34 10.1371/journal.ppat.0030034 17367208 PMC1828697

[B17] Weise T Global optimization algorithms–theory and application 2009 La Jolla, CA USA: Self-Published

[B18] Nair S Miller B Barends M Jaidee A Patel J Mayxay M Newton P Nosten F Ferdig MT Anderson TJ Adaptive copy number evolution in malaria parasites PLoS Genet 2008 4 e1000243 10.1371/journal.pgen.1000243 18974876 PMC2570623

[B19] Kidgell C Volkman SK Daily J Borevitz JO Plouffe D Zhou Y Johnson JR Le Roch K Sarr O Ndir O A systematic map of genetic variation in Plasmodium falciparum PLoS Pathog 2006 2 e57 10.1371/journal.ppat.0020057 16789840 PMC1480597

[B20] Wilson CM Serrano AE Wasley A Bogenschutz MP Shankar AH Wirth DF Amplification of a gene related to mammalian mdr genes in drug-resistant Plasmodium falciparum Science 1989 244 1184 1186 10.1126/science.2658061 2658061

[B21] Singh A Rosenthal PJ Selection of cysteine protease inhibitor-resistant malaria parasites is accompanied by amplification of falcipain genes and alteration in inhibitor transport J Biol Chem 2004 279 35236 35241 10.1074/jbc.M404235200 15192087

[B22] Medvedev P Fiume M Dzamba M Smith T Brudno M Detecting copy number variation with mated short reads Genome Res 2010 20 1613 1622 10.1101/gr.106344.110 20805290 PMC2963824

[B23] Yoon S Xuan Z Makarov V Ye K Sebat J Sensitive and accurate detection of copy number variants using read depth of coverage Genome Res 2009 19 1586 1592 10.1101/gr.092981.109 19657104 PMC2752127

[B24] Robinson T Campino SG Auburn S Assefa SA Polley SD Manske M MacInnis B Rockett KA Maslen GL Sanders M Drug-resistant genotypes and multi-clonality in Plasmodium falciparum analysed by direct genome sequencing from peripheral blood of malaria patients PLoS One 2011 6 e23204 10.1371/journal.pone.0023204 21853089 PMC3154926

[B25] Smith SW *The Scientist and Engineer’s Guide to Digital Signal Processing* 1999 2009 PO Box: California Technical Publishing

[B26] Ruby JG Bellare P DeRisi JL PRICE: Software for the targeted assembly of components of (Meta) Genomic sequence data G3 (Bethesda) 2013 3 865 880 2013 23550143 10.1534/g3.113.005967 PMC3656733

[B27] Li H Ruan J Durbin R Mapping short DNA sequencing reads and calling variants using mapping quality scores Genome Res 2008 18 1851 1858 10.1101/gr.078212.108 18714091 PMC2577856

[B28] Dharia NV Sidhu AB Cassera MB Westenberger SJ Bopp SE Eastman RT Plouffe D Batalov S Park DJ Volkman SK Use of high-density tiling microarrays to identify mutations globally and elucidate mechanisms of drug resistance in Plasmodium falciparum Genome Biol 2009 10 R21 10.1186/gb-2009-10-2-r21 19216790 PMC2688282

[B29] Neafsey DE Galinsky K Jiang RH Young L Sykes SM Saif S Gujja S Goldberg JM Young S Zeng Q Chapman SB Dash AP Anvikar AR Sutton PL Birren BW Escalante AA Barnwell JW Carlton JM The malaria parasite Plasmodium vivax exhibits greater genetic diversity than Plasmodium falciparum Nat Genet 2012 44 9 1046 50 10.1038/ng.2373 22863733 PMC3432710

[B30] Dharia NV Bright AT Westenberger SJ Barnes SW Batalov S Kuhen K Borboa R Federe GC McClean CM Vinetz JM Neyra V Llanos-Cuentas A Barnwell JW Walker JR Winzeler EA Whole-genome sequencing and microarray analysis of ex vivo Plasmodium vivax reveal selective pressure on putative drug resistance genes Proc Natl Acad Sci U S A 2010 107 46 20045 50 10.1073/pnas.1003776107 21037109 PMC2993397

[B31] Chen K Wallis JW McLellan MD Larson DE Kalicki JM Pohl CS McGrath SD Wendl MC Zhang Q Locke DP BreakDancer: an algorithm for high-resolution mapping of genomic structural variation Nat Methods 2009 6 677 681 10.1038/nmeth.1363 19668202 PMC3661775

