# Supplementary material for: Identification of pathogen genomic variants through an integrated pipeline
Source: BMC Bioinformatics. 2014 Mar 3;15:63. doi: 10.1186/1471-2105-15-63 (PMC3945619; doi:10.1186/1471-2105-15-63)
Supplement: Additional file 1: Table S1 — Presence (1) or absence (0) of potential recombination events in various isolates from the evolution experiments (Bopp et al, 2013) [6]. [file 1471-2105-15-63-S1.pdf]

Supplemental Material

Table 1 – Presence (1) or absence (0) of potential recombination events in various isolates from the evolution experiments (Bopp et al, 2013)

	R1a	R1a2	R1b	R1b2	R2a	R2b2	R3a	R3b	R4a	R4b	R5a	R5b	S1c
1_27000-8_1402000	0	1	0	0	0	0	0	0	0	0	0	0	0
1_32000-10_30000	0	1	0	0	0	0	0	0	0	0	0	0	0
1_35000-12_2243000	0	1	0	0	0	0	0	0	0	0	0	0	0
2_20000-12_10000	1	1	1	1	0	0	0	0	1	1	1	1	0
2_920000-4_39000	0	0	0	0	0	0	1	1	0	0	0	0	0
3_25000-9_11000	0	0	0	0	0	0	0	0	1	0	0	0	0
4_1190000-10_1656000	0	0	0	0	1	1	1	1	0	0	0	0	1
4_1193000-7_10000	0	0	0	0	0	0	0	0	0	0	0	1	0
6_12000-13_27000	0	0	0	0	1	1	1	1	0	0	0	0	0
6_1385000-9_17000	0	0	0	0	0	0	0	0	1	1	0	0	0
8_15000-12_2253000	0	1	0	0	0	0	0	0	0	0	0	0	0
9_64000-12_2199000	1	1	1	1	1	1	1	1	1	1	1	1	1
9_64000-10_55000	1	1	1	1	1	1	1	1	1	1	1	1	1
9_1502000-13_2863000	0	1	0	0	0	0	0	0	0	0	0	0	0
10_19000-8_1405000	0	0	0	1	0	0	0	0	0	0	0	0	0

Data 1 – PCR products of amplification across potential recombination events

>R1a2_01_08
ctaatatcatgaaagtaggtccacagagacctaatgtatgtcctaatgtaggtaatatagatcttagattaactaatataggtcttaagttaactaatataggtccaaagatacctaatgtaggtcctaatgtaggtaatataggtcgt
aagatcatgaaagtaggtccagagatacctaatgtacgtcctaatgtaggtaatataggtcttaggttaagcaatataggtcctaagatcatgaaagtaggtccagagatacctaatgtaggtcctaatgtaggtaatataggtct
taagaaactaacctaggtcttaaggtaactaatataggtcttaataaactaacctaggtcttaggttaactaatctaggtcctaagatcatgaaagtaggtacagagatacctaatgtaggtaacatagttcttaggttaactaatat
aggtcctaagatcatgtaagtagatccagagatacctaatgtaggtaatataggtcttaataaactaacctaggtcttaagatcatgaaagtagatccagagatacctaatataggtcttaataaactaacctaggtcttaggttaa
ctaatataggtcctaagatcatgaaagtagatccagagatacctaatataggtcctaatgtaggtaatataggtcttaagttaactaatataggtccaaagatacctaatgtaggtcctaaggtaatgaaagtaggtccaaagata
cctaatgtaggtaacatagttcttaggttaactaatataggtcctaagatcatgaaagtaggtcgagagatacctaatgtacgtcctaatgtaggtaatataggtcttaaagtaactaacgtaggccctaatatacctaatataggt
cctaagcttactaatttatgtcctataggtacgaatataggtcttaaaaagtagtgcacctatggtgcaccttcaaaaaaatagtggatctaaaccaagtgttcaaaaatagtgcaccttattaatgatattcgattaaaaaatttttca
tctagaaatttttttgataataatattacattagtatgataatcaaaaaaatacgtaacatgtttattatatactataaaacataatatatttaaattaactatataaattccaaaatgtcgtatttttattatttatagtatattgatataataaatat
ttgtcgttatattttatgtcatattgtcttgtaccatagaaatctcttgtaaaatttaatgtattaaaattataatataaatattaaataagtttttgatataaaatattaattaattatatattaacaaaatttataggttaattaatacgtatatata
aatatataattataaatatttaattttatcgtattaatttattgtattttattatctatgttatctctcttaaaagattgtatattattttaaatcgttgtttttattttatttgatttttcttttatatttataattttgtgtgtatttttctaataataaaataatt
aaaattttaattataatataaaatacttaatgttcttttaaacaacaatcaaggcgttaacattaatataatcttatatacaatgctataaataaactatttttagtactcccgaaaatagtatatatacaccaaatgggggtattgttgaca
acaagatgtgtaaatatacctataagcgtccttttatataacaataaataatttttaaatattatttatatatatatattataaataattttacatgaaattacaattaaatattcttacgagtatatacatatactctcttaatgaaattcttttcg
atttttttttttttttttttttttttttagaatttttttgtttatttaaaaatgaataaaatatattataaatataaaaaaatttataatgtacttataagagtattttatttaatatgcatactacttatatattaagataatatagtattataatattttttttat

>R1a2_01_10
atactgaagaaataaatggaacatcaactagtagtaggacaagacgaagtgcacgtggtggtagtgatcataaaggatatgaaaaaatttttttatgaaaaacttgaaggtgaatatagaaccgttgatggtttttggaattatta
aataaagaaaaagcatgccaagaagttaaagacagtgaaggaggaaaaattaattttagtgaaaaacacagtggtaatagtaatgatgaaacaaaaggaacattttatcgttcggaatattgccaaccatgtccttattgtgga
gtgagaaagacaaagggtaatcaatgggaaaataaaagtaaaaatgatcaatgcaatataaaactttataggcctaatcctggcgagtcaggtactcctattgaaatccttaaaagtggtgaaggagaaacagaaattgaaa
acaaattgaacgcgttctgtgctcaaacaaaaaatggtagtgttgttggtggcggtagtggtggtggtaacagtgatagtaaggaactttatgatgaatggcaatgttatgaatttaaacagctgacgacagatggtcaggagg
acgatgatgacgaggactatgacaaggatgtaagaactggaggcggattatgtatattggaaaaaaaaaaaagaagaaggtgaagccaaatctcaaaatgaacctgatgaaatccaaaagacatacaatgatttttttactat
tgggtggcacatatgttaaaagattctatacattggagaacaaaaaaacttgataagtgtataaataacactaataaatcaaaagcatgtaaaaacagtaataaatgtaataatgattgtggttgttttcaaaaatgggttgatcaa
aaaaaagaaaaggaatgggacgcaataaaacaacaatttcgcaagcaaaaagatattggagatgaaacaaattgtgatcctatcgtaactcttgaaggtgttttgcaaatagaatttttgaaaggcgattccgaatatggcaaa
aacgcacccacaagttggaagtgtgtcacaccaagtggtaacacgagtgacaccactgtcaaaagtggtgacaccaccggtggtagtatttgtgtgccacccaggagacgacgattatatgtcacaccactaacgagattg
acaggtggtgacagtaccacacaggcgtcacaggcgagtgaggtacagacacaagcacgtggtagtaacacggataagtcaccaggtagtagtgaggcagcacaaggtgacggcgtgtcgaaagacccacaaaag
gcactactcaaagcttttgttgagtctgcagcagttgaaaccttcttcctatgggatagatataaaaaaataaaagagaaggagaaaaaggaaaaaaagaaaacatatgaacaaatatatgaatcaaccgactatgacgatga
agaaaaagatccacaagaagaattaaaaaaaggaataatccctgatgagtttaagcgtcaaatgttttatacgttaggtgactacaaagatatattatacagtggtgatacggtgaatggtggtaatgaggacaaaataaaaaa
agctataaataactattttcaaaaaattcgtgaacaatcttctagtgataacaacccatctcctcgtagtgtcaaaacccccttcaactagtgacaaggaccctcaaacctggtggaatgcacacgccccttccatctggaatgcta
tggtatgtgctttaacatatgatacaaacagtggcggagagggcaaaaccacaactattacgcaggatcctaatttgaaaactgcactttgggacgaaaacggcaaaaaacccctcaaaaccaaataccaatatgatagtgt
cacaattggtgctagtggtgccaaaccccaaaccaaagccaaacccactggtggtgacaccccccctcacccaatttgtgttacgccccacctacttccgataccttgaagaatggggtcaaaatttttgtaaaaaacgaac

>R1a2_01_12
ttaaaggtattaaagaaaataaatggtcatgtggtaacgtatgtggttataatgtgtgtaaaccgaaaaaagttaatggggaaaaaggtagtggggaaaacaatgatcaaattataacaattagaggtttggttacacattgggt
acaaaatttttttagacgattataataaaattagaacaaaattaaagccatgtaggaataatggtgaggtatccaaatgtataaaagattgtgtgaaaaaatgggtagaaaaaaaaactgaagaatggccaaaaatacgagatcg
ttacttggaaccatataaaagtgatgatggctataacaaaaaatctttggttagaagttttatggagaccttgatacctctaatggatcttacaaatggtaaggaaaagattcaagaattaaataagttccttaggtcatatgaatgta
attgcgctgataactcacaacaaaaaggtgatacaccaaaagacatcgtagaatgtttgcttgaaaagcttgaagataaagcaaacaagtgtaaaacccaaactagtggtaccgactgtcacccctccaccccccttgaaga
tgacgatgaaccccttgaagaaacagaagaaaatactgtggaacaaccgaacatttgtccaacaaaacaaccacaaccagagaaagaagacggttgtgaagcagcaccaacaacagcagaagaaacgtcaccaacag

caactagtgaaggcacagagaaccaatcccctccacctcctcctccagcaccagcaccagcaccagcaccggcaccagaaaaatcacaaccaaaagaagacaaaaaagtggaaccacaacccaaaccacaaccaac
aaaccccccccccaaatttgttcaacaaccccgctgttatacccgccctcatgtcttctaccatcatgtggagtattggcatcggttttgctgcattcacttattttcttctaaaggtattatatatatatatgtatatgtggggatgtatat
atacaagtatatatacataaagacatatacatacacaaccatactaacctatatatatacctaaatgtacatacacaaaatatatataaaatatatatacataaatataactatttcttttttacttttttactttttttctttttctttatattttt
gtttaaataatttattttttttttttttttcctcttttataaaatttttattattttaattatattttatatttaaaactattttatttttacttttataatagtttttttttaattttttttaaaatatatatttttttactaatattttttttaaataatctttatt
ttatttttgttttcttcttattttttgtaattttttttttatatatagtattttattttttttttcttttttatataattttatttttatatatagtattttattttttttttcttttttatataattttttttttatatatagtattttattttttttttaattttttacaattttttttt
ttattcttttatttatttatttttttttttttttaatttttatttttttatttttttaatttttattttttttttttttttaatttttaattttttttttttttttttttctttttttttataaaataagtatgtgtatgtatatgtgtatattatatatatatataatatatgtattagttt
aaatgtatatgtatgtatatatgaatggatgtatgtatgtatatgtgtggatgtatatgtatgtatggagaaaataataaaatcttttttgatttgtgggacagctggaaaagtcacaataattataggggttttcactaatactatatgg
ctgttttgaaagtgggttatctatatatggaatatgatcaccatttatgtctccgtttgccatgtaaatggaacttcctctatcaccttgtctatcaccaatgtgactagtaatatcactataatgaagtagaa

>R1a2_02_12
cttcgacttcattactaacttactgatacattactacgacatcattactacttcttcaactacttcgttactactatgtgactactaacttactactactaacttaatacatcttactactactaacttactacatcttactactactaacttact
actaacatcatcactaaaatgactactaacatgactactaacgtcactactaacaacatcagtaacatgactacttacatgactacttacatgactactaacatgactactaacatgactactaccatcatcactaacatcatgagt
gacataactattaacatgactactaacttgactactaacttgactactaacttgactactaacattactactaacatgattactaacatcagtactaacatcataagtaacatgtctactaacatgactactaacatcatcacaactaac
atcataactaacatggtgactaacatgatcactaacatgactactaacaacatcactaacatcatgactaacattactacttacatgattactaacttgactactaacatgattactaacatgactactaacattactactaacatgat
cactaacatgactactaacatcatcactaacatgactaagaacattactaacatgactacttacatgactactaacatagtaactaacaacatcactaacatgactattaacatcatcactaacaacatcactatcatcactacttac
atgactactaacatcattactaacatgactactaacttgactactaacatcatcacaactaacatcatgactaacatcatcactaacaacatcactaacatcatgactaacattactacttacattactacttacatgactacttacatg
actacttacatgactacttacatgactacttacatgactacttacatgactacaaacatcattactaacatgactagtaacttaggtcttaacatgactaattaggtcttaacataactaatataggtctcaacataactagctaaggtc
atacaattactaacctaagtcattaaggtactaacttgggtcttactttcactgatataggtcttatgttaactaactaaggtcttaacttaactaacttgggtcttatcttcactgatataggtcttacctttactaatataggtcttgattta
actaaactgagaccttacattcactaatataggtcttaagtcatctaacttagaccttaatttcactaatatagatcttaaggttactcacataagtcattaaggtactaagttaggtcctcacataaccaatataggttctaacataaa
caatataggtcttaagtttactaacctaagtcattaaggtactaacttaggtcctaaggtaactaatatatatcttaactacagaaacttaggtcttaaggtacctaatataagtcattgaggtactaacttaggtcctaaggtaactag
tatagatcttaactacagaaacgtaggtcttaaggtaactagtataggtcctaatatcactaagttaggacctaactacagtaccataggtcttaacgtaactaatataggtcctaatatcacaaacataggtcttatgttaactaaa
ctaggttctaaccttactaactaaggtcataaaggtacaagtataggtcttaagttaactaagctatgtcctaagcttactaatttaagtcataaaggtacaatatagttcttaataaagtaatataggtcttactataactaatataggt
ccttttatcacaaaagtaggtcttaagttaattaaactaggtcctacgcttactaatgtaggtcttaaaggtactaataaaggtcctaatataactaatataggtcttagtattactagtataggtcctaatataagtaatatagatccta
aacttactaatttaggtcataaaggtactaatataggtcttaataaa

>R1a2_08_12
tcagtaactaagttcttaacatcactaacataggtcctaacattagtaatgtaggtcttaacttaactaactaaggtcttaacttaactaactcagaccttactttcactattattggtcttaattcatctaacttaggtcttaatttcaacta
acttagttcttaaattgagtaacatatgtcttaacttgactaacataggtcttactttcattaattgaggtcctaacattacatacttaggtcttacgttgactaactcaggtcatacttttactaacagaggtccttcttttactaacatagg
tcataaaattactaagctaagtcattaaggtactaacttaggttctatcttaactaatataggtcctaacttgactaacataggtccgaacttgactaacataggtcttaacttagctaacataggtcttaactcaactaactgaggtca
tatttcgactaatgtaggtcttacttttactaacttaggtcttacgttcactaatgtaggtcctaagttcagtaacttaggtcttaaggtcactaacataagtcattaaggtactaattttggtcttaacttaacaaatataggtcctaacat
tactagctttcgtcttaaggttactatcttaggtcttaaggttactatcttaggtcttaaggtaagtgatgtaggtcctaactacactaacgtaggtcttaacttaactaactcagaccttactttcactaatataggtcttaatttgactaa
cataggtcttaacttaactaaattaggtcttaacttagctaacatagatcttactttcactaacctaagtcttactttcactaacataggtcatacgttcactaacttaggtcttactttcactaatataggtcttaacttcagtaactaagg
tcatacttcaactaacataggtgttaagttaactaactgaggtcatatttctactaatgtaggttttactttcactaactgagaacgtaactaactaaggtcatacaattactaacctaagtcatgaaggtactaacttaggtcctaactt
aagtaatataggtcttaagttaactaactaaggtcttaacgtaactaactaaggtcatacaattactaaactaagtcattaaggtacttacttaggtcctaacgtaactaatataggtcttatagttactaactaaggtcttactctcact
gatataggtcttatggttactaactaaggtcctaacttaactaatataggtcataaaattactaagctaagtcattagggtactaacttaggtcttaccttcactgatatgcgtcttaacgtgactaacttaggtctaaacttgactaac
ataaggtcttatgattactaacttaggtcttaacttagctaacataggtcttcatttaactaaactgagaccttacattcactaatataggtcttaagtcatctaacttagaccttaatttcaactaacttaggtcatacttcgactaacata
ggtcctaagcttgctaatataggtcgtaagatcatgaaagtagatccagagatacctaatgtaggtaacatagttcttaggttaactaatataggtcctaagatcatgaaagtagatccagagatacctaatataggtcttaataaa
ctaacctaggtcttaataagctaacctaggtcctaagatcatgaaagtaggtccagagatacctaatgtaggtcctaatgtaggtaatataggtcttaggttaactaatataggtcctaagatcatgaaagtaggtccagagatac

ctaatgtaggtaacatagttcttaggttaactaatataggtcctaacatcatgaaagtaggtccaaagatacctaatataggtcctaaggtaatgaaagtacgtccagagatacctaatataggtcctaacattactaatgtacgtc
ttaccttcactcatataggtcttactttcacaaaagtagg

>R1a2_09_10
aaaatttttaaatagtaattgtattttttttttgttctaaaaattcttgtatgttataactttctctctactttatctacgtagttgcaaatattaatattattatttttataaaaatattttaacttattttaaaaaaatattaattattaaattatttaatta
tataattatcacaaaaaaaaatttaaatatttattataattataaaaaaaattcaaaaacaaaattaaaaaaaaaaaaaaaaaaaattaacgaaatgagaaattatatatactattacacaatatattgtattgtttatatatagcgttttatc
ttatacaaatatattatatttattattattattatatatatatttcttgaaatagtaatagtatttcacaacttatcatacattaattgcattattacatattgtaacaaaatatataattactatatattttaataattattttgtaataaattatattatc
gcatatatattaaaacaataacacaacattgtatatactattaaaaaacaaggaatcatatatatatattttatactaaattagaaacattaaaaagaaattcttaatattactaaaaaaaaatgaggttatttcctataatgaggataat
agtacatatgaatacaaaaaaaaaaaaaataaaaaaaaacaaaacaataattagaaaaaaatgaagaagcatgaacgaaaaaagaacaacataaatataaaaaataaaattattattatatcatgaatctggtgataccatatatt
atttttatattagaaaaagaaacttttaacgatatgttaaataaataacctatagatgcatggatactattaaaaatgtgttttgaaattgtttatggatactcacaaataattatatattataatataataagtatatattacatatcttttattg
tatattctatataaagaatttaatgaatatatattacattaagatttcatctaatatagtaaaattttaaataatcttctatataaagccacaaatattttaccttatcttcaaatccttctttagttataccaaaaacatgattccatataccata
aaaaccttcattagatggacattcttaattatctattatattgtgtaattcttccagggttaactattttgataaatcattataatttatattattacatttttcctcagttgtaactccattatattttatatcatcttcgcaaatacaaccttctga
actaaaaggtatattattattttttttcatctacagtattttttaatttaaaaatttcattgttacttcttaatcctgaatattctacatcttttaactcagataaatttcttctatggataaaataaatcccatcattcatcgtttcactttttcacata
tacttatactctgaagaaataataattattataaaaaggtcaatataggttataaaaataaattgatattataaaaatattcacatttattaacttacgtttaatattactactaatactattccaacaattgataacaaatttaaaaatatttg
tctaacggaacaaattttcagtgtatttttcatattttcttcttcagaataatatcctacataatggaatttcatcttaatcataatattattataattatattatattttaaaaaattaatattatttattttttaaaatatattaaaattgtatgatgaa
taatgtttttttatgaatactatctgcattattcttttaatgtattaattgtaattcacaaaaatatatacatatatacacatatatatatatatatatataataaattattttataatataattaataatatttatttattatttttatattttgtgttca
ggcaaaaatattattataaatatatttatagttttataaaagttaataatataatgatatatatattttttttctaaatattatagtactcttaataatattaacataa

>R1a2_09_12
aaacaaaaaaaaaaaattttttaaatagtaattgtattttttttttgttctaaaaattcttgtatgttataactttctctctactttatctacgtagttgcaaatattaatattattatttttataaaaatattttaacttattttaaaaaaatattaattat
taaattatttaattatataattatcacaaaaaaaaatttaaatatttattataattataaaaaaaattcaaaaacaaaattaaaaaaaaaaaaaaaaaaaattaacgaaatgagaaattatatatactattacacaatatattgtattgtttat
atatagcgttttatcttatacaaatatattatatttattattattattatatatatatttcttgaaatagtaatagtatttcacaacttatcatacattaattgcattattacatattgtaacaaaatatataattactatatattttaataattattttgta
ataaattatattatcgcatatatattaaaacaataacacaacattgtatatactattaaaaaacaaggaatcatatatatatattttatactaaattagaaacattaaaaagaaattcttaatattactaaaaaaaaatgaggttatttccta
taatgaggataatagtacatatgaatacaaaaaaaaaaaaaataaaaaaaaacaaaacaataattagaaaaaaatgaagaagcatgaacgaaaaaagaacaacataaatataaaaaataaaattattattatatcatgaatctgg
tgataccatatattatttttatattagaaaaagaaacttttaacgatatgttaaataaataacctatagatgcatggatactattaaaaatgtgttttgaaattgtttatggatactcacaaataattatatattataatataataagtatatatt
acatatcttttattgtatattctatataaagaatttaatgaatatatattacattaagatttcatctaattagtaatattaaatataataatgtaaaaaagtatcatgattgtggtaatataaaatttactagaaatgataatattggaagacttg
actttcgatataataaaaaaattaagttggaaaggtaacaatggggaattaggacttacaagatgcgtaaaatgaagtctatttaaagaggatatatatagtatttaaagatgtaagagacaaagtgaaaacagtgtataataatat
gtgatttgtttttatttattatggttttctagttatttttaatatatatatatatttaactataatatttttataagtgtaaataatagaatgcaaatcatgattctgatttttataaggtctttaacaattgtttaaactttataagagaatagtcttgt
ttaaaagcaacataaaaattacaccaaaacgggtcttattataagatgtgttaccgtaaagtacaatatgaataataataataaaatataaaattttttattataatacataaaaattttatataatttttcatttaacaaaatatgaatata
aagaatgtaataagaaaattacataattaatacaaagttttcttatatatttatatatttattacttaaaatttatatatatatataaataataaagttataaaatagaactacatttatataataatacttatataaatataataattttttaattt
tatattaatatttatataaccaaaacatttataatattgtgagaagtattatgattgtatgttttattgtgaataaaactttaagtactgtaaaaatatattattaaattaatttaattttttttattattattagacttaatattatgtatatgtatatat
acacatagtattaattttactttgtctaatataattaatatttttatatataaataatagattaaaatgttgtaagacatattatataatttatttttattttttttatgtgttaaaaatataaagataa

>R1b2_10_08
aaggtcatacttcgactaacataggtcttacattaactaacataggtcctaacattagtaatgtaggtcttactttcactaactaacgtcttaccttcactaacataggtcttaacatgactaacataggtcttaggttgactaacttag
ctcttacttttactaactaaggtcttaacttaactaactcagaccttacttatactaatataagtcctattttaactaacataaggtcttaacttgactaacataggtcctaacttgactaacataggtcttaacgtcagtaactaaggtctt
aacgtaactaactaaggtcttaacataactaagtaaggtcatacttcgactaacctaggtcttactttcactaacatagatcttaagtttactaactaaggtcttactttcactcatataagtcttatggtaactaactaaggtcctaact
taactaatataggtcataagattactaacctaagtcattaaggtactaactcaggtcttacattcactgatataggtcttaaggttactcacataagtcattaaggtactaacttaagtcctaacttaactaatataggtcttactttcac
aaacatagcccttaacttaactaactaaggtcctaacttaactaacataggtcttagcttgactgacataggtcttaacttgactaacataggtcttagcttgactgacataggtcttaactttactaagtgtggttctacgattactaa

cttaggtcatacgtctactaacataagtctaatcttgactaactaaggtcttacattcactgatatcggtcttaacttgactaactaaggtcttaacttaactaactcagaccttacttatactaatataggtcctattttaactaacataa
ggtcttaacttgactaacataggtcctaatttgactaacataggtcttaacttgactagcataggtgctaacattagtaatgtaggtcttactttaggtcttactttcactcatataagtcttatggtaactaactaaggtcttaccttcact
gatataggtcttactttatctaacataggtattacttttactaacttaggtcttaaattcagtaactaaggtcatatttcgactaatatgggtcttaacttcactatctctcgtcctactattactaacttaagtcttactttgcctaggtgagtt
ccttcttttactaacatagtcttactttcactaacttaggtcttaaattgagaaactaaggtcatacttcgactaacgtgggtcttacattaactaactcaggtcttactttcactaacatagttcataacttaactaatctaggtcttaaatt
gaataactaaggtcttacttttactaacatagatcttactttcattaattgaggtcttgacttagctaacataggtcttactttcactaacttaggtcttaacttaagtaatctaggtatgaacttcaataactaaggtcttacttgaactaa
ctaaagtcttacttttactaacttaggtcatacttcgactaacataggtcttacattaactaacataggtcttaacttcactaacataggtcttaacttaactaatataggtcctaacataactaacataggtcttactttcactaacttag
gtcttatatcaactaaataaggtcttttcttcactgatataggtcttatgattactaactaaagtcctaagttaactaatataggtcataaggttactaacctaagtcattaaggtactcacttgtgtcttaccttcactgatataggtcttat
gattactaactaaggtcttaacttaactaatataggtcataagattactaacataagtcattaaggtactaacttaggtcctaaggtaactaatagaggtcctaactacagcaacataggtctataggtaagtaatataggtcctaac
attactgatgtaggtcttaccttca

>R1a2_09_13
cgtttggcacgtgtgggttcggcacgttcactctcactgccagtggtttcaccaggtgtgttgttgtcaccaccactagtggtaggaacacatttccagccgtagtttttgcctttcacatattttaaattacatgcatcttggagattt
tccctgttttctagtgcggtttttactatatcacacaccttggggtcttctactgttgtttcttttgttgttaccacatccccctcctttgccgtttccacttccgcctcctggtcttccgcgtctttgcgatcgaggtcgtcgtcgtcttcttt
gtcttcttcctcttcttcttcttcttcttcttcttcctctggttgaacttcagaacgtggttgtccaatttttgcaacaccatctccgccgggtggttgtggttgcgtttcttgacagtctttgcatttttggcttcgtctttttcgtagtcga
tcaatttatccattatagtttctgtccattggcaccagctggttcttgatttttttttcaatgatttcgcgaatgtattttagttcctctgcctcctcttcatccagactattttcggtatcttgtgcggaaccgtcttcggattttctttataaa
attctaatttcaaaacttcttgaagaacaaaatcaggagattctatttctttgcctaacattccacctgcgatattttctccttgcttgcgaaaatcttgcttgcgaaattgttgttttattcggtcccattcgtcttttttttgtttaatccatttt
tgaaaacattcacaatcattattacattgttggtttccacattttattctgttaccattttgtaaacatctctgaagttttttttccaatatatagaatcttttaacatatgtgccacccaaaaattaaaaaaatcattgtatgtcttttggatttc
atcagggtcattatcagtctttttttcttttttttttttaatatgcacaatccgcctgcattacatattgagcttcatagcatttccatggttcacacaaagaagaatcactattaccaccaccacaatcaccactagaaccactagcatt
accactaccaccaccaccagcactaccattttgtgttaggcaaaacttgtcaattttacttttttatctcatttcctttatcaccgaaggaaaggacgttgatatcaataggtttggcaccatcttcaatcttataatgtttatcactatcac
agtcgccatttttttctctttccattgattatctttacgtttgaccccacatccgggacagggttggcaatattccgaatgataaaatgttcccttattattaatatttttatcaaaacgataatcacatttattagaaaaatcaattatttctt
cttgctcattaatacctttacattcatttctttatttaataattttaaaaaatcatcaacggttccatagttatttttttctttcagtttttttataaaatttactttcatatccttcatacttgatagttgcagctcgcgtttcctactactacccttaa
caccaccactcccaccacaactaccgctatttgatatttctgtttcgtatttgtttctttgttttagaaattcttttttctggttatctatccatttttcatacagacgacaccaaacagaacatttagtgcattcactacctataacaagttta
cctttttatatatagtttttgtgcaatcgtagccattaccactacaatattttactgtttcatcacgacaatttctttttaacatcttttaattttttttttttttacgacagaaatcttctgcccattcctcgaaccatctcaaatattgaggcaca
tagtcaaaatatgtggggacaatatttacgtcgtcatttgccttgccggcctttgacttgtcgtgacctggctggtcgccgtcacaccggcagtatttattagcttgagactgactctttccatcact

>R3a_02_04
tgtatcgcacgtttttactgttccgcaaactccattaccaccaccccccactttcctctttgcattttgtttttttgtttaacatatgcactttttttgttccccaaattctattttttttcttctatccatttttttatatttctacattctcttccacaacct
ggacattttaaatccgcaaaaactcctatatttgatatatcttctattttacactcttccccataaccactacatttgtaatcagcactatccactttacaatccactttaatttgtgccaacttcttttttcctttctttacaaaatgtttcgccc
cattcttcaaggtatcggaaataaggggggcgtaacacaaagtgggataggaatgtgggggtgttatcactaggggctgtggctttagtgtctttggcttctgtgtcactattttcgtcgagtttgacggtgttatattcgtatttttc
tttgtatttgccactagttgtgccaggggtggtgccaaaaaatttctcgtacacttcattatcctttactattttttttcgtcattttctttgtatgttagtgcacatatcattccttcccaaatggaaggggcgtgttctttccacaaggttt
cacgtgttaaactactattttgttgttgttggccggggcgttgagggttggggacactagcagcttgtttgagactatttatatgttcatctatagctttttgtattttcttcatttcatcctgtttgtcaccacttgcttcaatcacaatattg
ttgttgttactaccatttgtgttaccactgtcacttgtgttaccacctcgtaccaaaatatctctataatcacctaatgtataaaacatctgacgtttaaactcctcagggatatctcctttttctaatttcttttgggggtctttgtcttcgtc
gtcgacgtttccactgtttccgtcgagggcacctactaatccactttctcgttgttgtttttttcttttttggttgtttcgttccaaagtttctccggattttcaatcttttgtggtttttttacgacgcctttgaggccattgtcgtcatctttatatg
ttaatgcgcaaaccattccatgccaaacatgttcggcgattctatcccacaaggattgacggtcattgacactattttgggggccacgagtttctttgttaccactagttggaagaatttgttctattttctcttgtattttctccattttct
gtttttcatccttgttaccactcgcattaagtattatgttgttgccaccgtttttgtcgtcagcaacacctcgtaccaaaatatctctataatcacctaaagtataaaacattaatctcaagaaatcgaggggtatggtaccactttttttgt
aattgtgtttgggggttggagtcatcaccaccaaggttgaggctagcacctgtgccgagtagtaatccatttcgttgtagttccgccttcttttgtgcctcccactctttttttatatctatgccataagaaaaaaagtctctatagctgcc
gtctctataaaccacttacgtaatgactcggtggtggtgatgtcgtcgccgccgttaccttgtgacacatcactcgcccgtgatgactcactacccccctgtggtgactcactagacgtgccagcacttgtcaacttggttaacc
cacccacgtatagtcgtcgcctcctgggtggcacacaaatggcacccttatcaccacttttgccagtggcaccactactaccggcagtggcaacactttttttcaccacttgatacacatttccaattggggaatctttcctttcca
cctggaccgtattttagtttacatgcatcagtgagattgtcgttgtttgtgagtgcgtttttcactgtgtcgcaaacgtctagtgatgtagtcgtatctgttgccttctttgcctcttcctcctccccctccctgtccgtgtccttcaccgg

ctgctccaccgtctctgtctcatccccttctgtggtgtccaccgtctccccctcctcgacttcgtcgcctgcgtcttcttcttcgtcttcttcttcttcttcgtcctcaagtgtagtggtttctccggtttcggagggggcaccaggacct
ccagcgccgggtggttttggttgcgtttcttcgcatttttctttgtgtgtttttaggcagttattggcatcgtctccttcatgttgtagtaatttatcaattgtggtcttcttttgattgtcggcaacaacaacaacaactgcttcttctgcttg
tttttttttttttcctcatccaacagttctttaattccttttaattcctttacatccccataaccatctttaatatcttggaaaagttgttctaattccaaagccaattcaagaacaacatcagcagaatccattagtccgctgaacatgtcaa
ttccgctattttctcgtttattcttaaaagcttcttgcgttttaaaatgtttttttatattttcccattcttctttcttttttccaatccattctttaaaacaaccacaatcatccttacattcatttctacatttctttcgcttaggattatttatacaact
gttaacttttcctctccaatacatagaatcgttcaaaaaacgtcctatccaaaagtaaaaaaaatcatggaatgtcttttggaattgctcaggttcatttgaagaattatttctactttcatgtttttttgttttccaatatacataatccgcct
gctttttttaccttgtctacatcttcctcgtcttcatcctcttcctctccatttttatcatctttaactttctgtacctcgttataacatttccattcttcatacagttccttactattactcttactaccactaccatcagcgccaccgcctctagc
aacactatttgttgtatcaccatttgtttgatcgcaaaacgcttttaattttgtttcaatttctttttgtttttcac

>R3a_04_10
taagttagtaaaggtgagacctaagttagaagaagtatgacctaagttagtaaaagtatgaccttacttagttaagttaggacctaagttagttatgttaagacctaagttagttaagttaagaccttagttagttatgttaaaaccta
agttagttaacttcaggcctacgttagtgaaagtaagacctatatgagtgaaattaagacctgtgttagtgaaagtaagaccttagttcgttaatctaggacctaagatagttaagttaagacctaagttagtagaagtaagaccta
agttagaagaagtatgacctaagttagtgaaagtaagaacttagttagtcaagttaagaccttagttagtcaagttaagaccttagttagttacgttaagaccttagttagtaaaagtaagaccttagttcgttaatgtaagaactta
gttagtcaagttaggacctgtattagttaccttaagacctatgttagtgaagttaggacctataatggttaagttaagacctatgttagtcaagttaagacctatgttagtaacagaaggacctcacttagtgaacgtaagaccttag
ttagtcgaagtatgacctatgttagtaaaagtaagacctatgttagtcaacgtaagaccttagttagtaaaagtatgaccttagttagtaaaagtaagacctatgttagtaaaagtatgacctatgttagtaatagtaggaccagagt
tagttaagataagtcctatgttagctaagttaagacctatgttagtcaagttaagacctaagttagtcaagttaagacctacgttagtcaagttaagacctaagttagtaaaagtaagaccttagttactgaacataagacataagtt
agtaaaagtaagacctaagttagtaaaagtaagacttaagttagtgaaagtaagacctatatcagtgaaggtaaaaccttagttagtcgaagtatgacctatgttagtaaacgtaagttagtaatcataagacctatatcagtgaa
tgtaagacccacgttagtcgaagtatgacctaagttagtaatcgcagaaccagagttagtaaagttaagacctatataagtcgaagtgtgaccttagttagttaacttaacacctatgttagttgaagtatgacctttcttacttaata
taagaccttagttagtaaacgtaagacctaagttagtaaaagtatgacctaagttagtgaaagtaagacctatattagttacgttaggaccaaagatagtaccttaatgacttagcttagtaattgtatgacgttagttagttacgtta
agaccttagttagtcgaagtatgaccttagttagtcaacgtaagacctatgttagtcaagttaggacctatgttagtcaaattaggacctatgttagttatgttaggacctaagttagtaaaagtaagacctaagttagtaccttaag
acctatgttagtaatattaggacctatattacttacctatagacctatgttgctgtagttaggacctgtattagttaccttaatatctacgttagttacattaagacctacgctactgtagttaggacctatatttgttaagttaagaccaaa
cttagtaccttaatgacttatgttagttacgttaagacctatgttagttacgttaagacctatgttagtcaagttaagacctatgttagtaaaagtaagaccttagttattgaagttaggacctatgttagtcaaattaggacctatgttag
ttatgttaggacctatactagttaccttaagacctacataacttaccttaagatctacattacttacctaagacctacattacataccttaagacctacattacttaccttaagacctacattacttaccttaagacctacattacttacatt
aagacctacgttactgtagttaggacctatatttcttaagttaagacctacactaataatgtta

>R3a_06_13
agtgtgtttggatgtatatatatatatatatgttcatatatatgtgtttctgtatatatatgtatgtgtgggtatatttggatatatatatatggatttacatgtatgtgttttatatatattttatatatatgtatttatattaaaaaagaaaaaaatg
aaaaaaagaaaaaaaaaaaatttattaaaataaaaaaaagaagagaaagattttaaaaataataaaaattataataaaaatataaattttgataaaataaaaaatgaaaaattttatcaaaaaaaaaattaaaaaaaaattatattaata
aaaataatgattagaaaaaaatttattagaaataaaataaaaacaaatttattaaataaaaacaaaaaaaaaaaaaaaaaatgttaaaaaaaaaatatatatcataaaataaaaagaaaagggaaaaaatgttaaaaaaaaaaaa
aattaaaaaattaaaaaatttaaaaaaaaattaaaaaatttatttaaataaaaaaaaaattaaataaaataaaattaaaaaaaattaaaattaaaaaaaataaaaaaatttaattaaataaaaaaaaaaattaaaaaaattaaaattaaaaa
aaataaaaaaatttaattaaataaaaaaaaaataataaataaaaaaaaatttaattaaataaaaaaaataaaataaaattaaatacatgcacatatacatatacgcatacatatacacatacacataaatatatatattatatatatgtacc
cataactacgtacacatttacacatacatatatatatataccaataactacatacatacatacatacatacacacctaaatacatatatatacatacacatatatgttcatttttttttttagaaaaaaactaaacgccttgtggaccttttc
agtgttattaatatccccaaaagtgattatgatataccgacaaaactttcacccaatagatatataccttatactagtggtaaatacagaggcaaacgcaatgaatataggaggtaatgttttagatgatggcataaatgaaaaac
cttttattatgtctattcatgatagaaatttatatagtggagaagaatatagttataatgttaatatggttaatagtatggatgatattccaattaatcgtgataataatgtttatagtggtatagatttaattaatgacacgttaagtggtaa
caaacatattgatatatatgatgaagtgctaaaaagaaaagaaaacgaattatttgggacaaatcatacgaaaaaaaatacatcaaccaatagtgttgcaaaaaatacaaatagtgatcctatactcaatcaaataaatttgtttca
tacatggttagatagacatagagatatgtgcgaaaagttgaaaaatgataatgagcggttagccaaattaaaagaagagtgggaaaatgagacacatagtggtaacactcaccctagtgatagtaacaaaacgttaaatact
gacgtttctatacaaatacatatggataatcctaaacctataaatcaatttactaatatggatactatcttggaagatctggaaaaatataatgaaccttattatgatgtacaagatgatatttattatgatgtaaatgatcatgatgcat
caactgtggatagtaatgctgtgaatgttcctagcaaagtacaaattgaaatggatgtaaatactaaattggtgaaagaaaaatatcctatagcagatgtatgggatatataatatatacgtatgaatgtatatttgtttgatttttattt
atttattatttttttttatttgtgtattatattatattttatatttgttgatgtaatattatatttgatttgtgtattatattatattttttatttgttgatgttgtaatatatttgatttgtgtatgatattatattttatatttgttcatgttatattatattttatatttg
tgaacttattttttttttttttttgtatttaat

>R4a_03_09
taacgtagatgctaacataaccaatataggtcctaactacactaacttaggtcttgcctttactaacttaggtcttactttcactaacataggtcttactttcattcatataggtcttatggtaactaactaaggtcttaccttcactgatat
aggtcataaaattactaagctaagtcattaaggtactaacttaggtcttaccttcactaacataggtcttattttcactaactgaggtcttactttcactcatataggtcttaagtttactaacataagtcattaaggtactaacttaggtc
ctaaggtaactaatatagatcttaactacagaaatttaggtcttaaggtaactaatataagtcctaactacagcaacataggcctataggtaagtaatataggtcctaacattacatacttaggtcttaaggtaactaatgtaggtctt
aaggtaactaatctaggtcttaaggtaactaacttaggtcctaacattatatacttaggtcttaatgtaactaacgtaggtcttaaggtaactaatagaggtcctaactacactaacttaggtcttaaagtaacaaatataggtcctaa
cattactagctttcgtcttagggttactaacttaggtcttgaccggagtaatgtaggtcctaatattactagctttcgtcttaaggttactaacttaggtcttgactggagtaatgtaggtcctaacattaccaatgtacgtcttactttca
ctaactcaggtcgtacgtttactaacgtaggtcttactttcactaacatgggtcataacttgactaactaaggtcttactttcactaactaaggtcagacttcgactaacttaggtcatacttttactaacttaggtcttaaattgagtaa
ctaaggtcatacttcgactaacgtaggtcttacttttactaacttaggtcttactttcactaactaaggtcatacttctactaactaaggtcttaaacataggtcttacttttactaacttaggtcttaacttaactaacataggtcataaaa
ttactaagctaagtcattaaggtactaacttaggtcttaacttgactaacataggtcttaagttaactaactaaggtcacacttcgacttatataggtcttaactttactaactctggttctgcgattactaacttaggtcatacttcgact
aactgaggtcttaacttaactaatctaggtcttaacttcagtaactaaggtcatacaattactaacctaagtcattaaggtactatcttaggtattaacttaggtcataacttaactaatatagctcttaagtttactcacataagtcatta
aggtactaagtttggtcttagcgtaacaaatataggtcctaacataaacaatataggtcataagtttactaacataagtcattaaggtactaacgtaggtcctaaggtaactaatagaggccctaactacagcaacataggtctat
aggtaagtaatataggtcttactttcactaactaaggtcttacgttcactgatatacgtcttaacttaactaacataggtcctaacttgactaacataggtcttaggttgactaacataggtcataaaattactaagctaagtcattaag
gtacaaacttaggtcttacattcactgatataggtcttatgattactaacttaggtcttatggttactaacttaggtcttaaggtcactaacataagtcattaaggtactaattttggtcttaacttaacaaatataggtcctaactacagc
aacataggtctataggtaagtaatataggtcttacttttactaacataggtcttactttcattaattgaggtcctaacattacatacttaggtcatacgttgactaacataggtcctactttcactaacttaggtcttactttcactaactga
ggtcatacttcgactaacataggtcttaactcaactaa

>R4a_06_09
aaggcatatgtattctctcaaaatatatatatataacagcatacatattcttaaaccaaaaatatacatatatatatatataataattctcggttactgcatgtgtccgtgcacgatttcgatttttgggtatcttccatttcgattttcatatt
tttcgcacttacattgctacggatttcgttacttccatatcttagtataggctttatctcttaattctactttggattattatttgttcatctttatgacactgcaacatgaatatcacatatgtatgaaattaccttgatgaacagatatgttcttt
aatattatacttccacatacatatacatatatatatttatacttgcatggcggatgtatatatacttatcatatataattagatacaatatatagatcctcacgttctatgagaatacagatctttatatagtagctatagatcctacacatat
ataacaataaaaccatatgagatacaaaacatacataaataataatataaacaaaatatatacctactcaacaattatagatataatccatatataaaccctaacccctaaagttataccctttaaccctgagagatgggaacccctaa
aatttttaaaccctaaaagtatatccttaaccctggtacatgggaacccctaaaatttttaaacccaaaaaagtatatccttaaccctggtacatgggaaccctaaaccctaaagactagatccttttattttaagactaggtccttttt
ttttaagactagatccttttattttgagactaggtcctctttttttaagactacatccacttttttttaagactagatccactattttttttttttgagacaaaaaaattttttttttttttttttttttttctttttttttaagatatatattttttttatatactttta
agacatagaaaaaaaaaatttttttttttttttttttttttttttttttaaaatgtacatttctattaacgtggtttcattttacaacgtggagatacgtgtgttaaaattatgcgtggagatacgtgtgttaaaattatgcgtggagatacgtgt
gttaaaattatgcgtggagatacgtgtgttaaaattatgcgtggagatacgtgtgttaaaattatgcgtggagatacgtgtgttaaaattatgcgtggagatacgtgtgttaaaattatgcgtggagatacgtgtgttaaaattatgc
gtggatatacgtgtgttaaaaaaactggtgacccaaataccatataggtgataaaaatttgtgatccaaaacccattttcgttgtgtacacttcttggtggtgaacacaccatactggtacttggtcacaaatagattgtgaaccaa
aatccattttcgtgatgataaaacacttcttggtggtgaacacaccatattggtggtgtacacaacataatggtgcttcgtcacaaatagattgtgaagcaaatgtctatgataaaaatttcacgtggtatccacgtgataaaaactt
gtgaaccaaatctaggtgataaaaaacttccacgtggtatccacgtgatgaaaaattctgccttaaatcattacgattctggatataagcatggatatcctgtatttaatcattcgaattctggattcaatcactggaatgctgaatat
aatcatgtatatactggattgaatcattcgaattctggatataatccatatataatcccggatgcaatcaatgacattctggatataatggatgtaatcctgcatgtaatccatagagacctggatgaaagtactagaatcctggata
taatcaattggattctggatccaatcaaataattctggatatagtcatggacatattgtatttaatcattcgaattctggatataatcaattgaattctggatccaatcaaataattcttgatataatcaatacaatcctggatgtaattcat
agagacctgcatgaaa

>R5b_04_07
aagttaagacctaagttagtgaacttaagacctaagttactgaagttaagacataagttagtaaaagtaacacctaagttagtaaaagtaagatctaagttagaagaagtatgacctcagttagtcgaagtctgaccttagttagt
caagttaagaccttagttagtcaagttaagaccttatgttagttaaaataggacctatattagtataagtaaggtctgagttagttaagttaagacctaagttactgaagttaagaactatattagtgaaattaaggtctaagttagatg
acttaagacctatattagtgaatgtaagatctcagtttagttaaatgaagacctaagttagtcaagttatgtactatgttagtgaaagtaagacctatgttagcaaaagtaagacctaagttagtagaagtaagacctaagttagtgt
agttaggacctatattggttaccttaagacttacgttactgtattgaggacctatattacttaccttaagacctaagttagtaaccataagacctaagttagtaaccataagacctaagttactaatcataagatctaagttagtaatca
taagacctatatgagtgaaagtaagacctacactaataatgttaggacctatgttagtcaaattaggacctatgttagtccaagtaagacctaagttagttaagttaagatcttagttagtgaagttaaaacctaagttagtaaaagt

atgaccttacttagttaagttaagacctgagttagttaaggtaagacctaagttagtcaaccaaagacctatgttagtcatgttcttactgatgttgttagtagtgatgttagtagtcaagttagtaatcatgttagtagtcaagttagta
atcatgttagtgatgttgttacttactatgttagttatgatgttagtaataatgttacttgtgatgatgttagtagtaatgtcagtgatgatgttagtgatgatgttatcaagttagtattcatgttacttatgatgttagtagtcatgtaagtag
tcatgttattgatgttgttagtgatgatgttagtagtcatgttagtgatcatgttagtcaccatgttagttaggatgttgttagtcactatgttagtagtcaagttagtattcatgtaagtagtaatgttagtagtcatgtaagtagtcatgta
agtagtaatgttagtagtcatgttagtagacatgttacttatgatgttagtagttatgtcactcatgatgttagtagttatgtcactcatgatgttagtgatgttgttagtagtcatgttcttagtcatgatgttcgaagtaatgttagtattca
tgtaagtaatcatgtaagtgatgttgttagtagtcatgtgagtgatgttgttagtagtcatgttggtgatgttgttatttactatgttagaagtcatgtaagtagtcatgttagtgatgttgttagtagtcatgttagtgatgatgttagtaat
gatgttagtgatgttgttagttactatgttagtgatgatgttagtattcatgtgagtgatgttgttagtagtgatgatagtgatgatgttaatagtcatgttagtagtaatgttagtagtcatgtaagtagtcatgttagtagtcatgtaagt
agtcatgtaagtagtcatgttagtgatgttgttaatgatgatgttagtagtcatgttggtgatgttgttatttactatgttagtgatgatgttagtagtcatattagtgatcatgttagtagtaatgttggtagtagtcatgttagtaatgatgt
tagtagtaatgttagtagtcatgtaagtgatgttgttagtagtcatgtaagtgatgttgttagtagtcatgttagtgatgttgttagtagtcaagttagtagttatgttagtagtcaagttagtagtcaagttagtagtcaagttagtagtc
atgttagtaatgatgttagttgtgatgatg
